# TREM2 Alzheimer’s variant R47H causes similar transcriptional dysregulation to knockout, yet only subtle functional phenotypes in human iPSC-derived macrophages

**DOI:** 10.1186/s13195-020-00709-z

**Published:** 2020-11-16

**Authors:** Hazel Hall-Roberts, Devika Agarwal, Juliane Obst, Thomas B. Smith, Jimena Monzón-Sandoval, Elena Di Daniel, Caleb Webber, William S. James, Emma Mead, John B. Davis, Sally A. Cowley

**Affiliations:** 1 James Martin Stem Cell Facility, Sir William Dunn School of Pathology, University of Oxford, Oxford, OX1 3RE UK; 2 Nuffield Department of Medicine Research Building, Alzheimer’s Research UK Oxford Drug Discovery Institute, University of Oxford, Oxford, OX3 7FZ UK; 3 Weatherall Institute of Molecular Medicine, University of Oxford, Oxford, OX3 9DS UK; 4 UK Dementia Research Institute, Cardiff University, Cardiff, CF24 4HQ UK

**Keywords:** TREM2, R47H, Alzheimer’s disease, iPSC, Human pluripotent stem cells, Macrophages, Microglia, Phagocytosis, Inflammation, Synaptosome

## Abstract

**Background:**

*TREM2* is a microglial cell surface receptor, with risk mutations linked to Alzheimer’s disease (AD), including R47H. TREM2 signalling via SYK aids phagocytosis, chemotaxis, survival, and changes to microglial activation state. In AD mouse models, knockout (KO) of TREM2 impairs microglial clustering around amyloid and prevents microglial activation. The R47H mutation is proposed to reduce TREM2 ligand binding. We investigated cell phenotypes of the R47H mutant and TREM2 KO in a model of human microglia, and compared their transcriptional signatures, to determine the mechanism by which R47H TREM2 disrupts function.

**Methods:**

We generated human microglia-like iPSC-macrophages (pMac) from isogenic induced pluripotent stem cell (iPSC) lines, with homozygous R47H mutation or *TREM2* knockout (KO). We firstly validated the effect of the R47H mutant on TREM2 surface and subcellular localization in pMac. To assess microglial phenotypic function, we measured phagocytosis of dead neurons, cell morphology, directed migration, survival, and LPS-induced inflammation. We performed bulk RNA-seq, comparing significant differentially expressed genes (DEGs; *p* < 0.05) between the R47H and KO versus WT, and bioinformatically predicted potential upstream regulators of TREM2-mediated gene expression.

**Results:**

R47H modified surface expression and shedding of TREM2, but did not impair TREM2-mediated signalling, or gross phenotypes that were dysregulated in the TREM2 KO (phagocytosis, motility, survival). However, altered gene expression in the R47H TREM2 pMac overlapped by 90% with the TREM2 KO and was characterised by dysregulation of genes involved with immunity, proliferation, activation, chemotaxis, and adhesion. Downregulated mediators of ECM adhesion included the vitronectin receptor αVβ3, and consequently, R47H TREM2 pMac adhered weakly to vitronectin compared with WT pMac. To counteract these transcriptional defects, we investigated TGFβ1, as a candidate upstream regulator. TGFβ1 failed to rescue vitronectin adhesion of pMac, although it improved αVβ3 expression.

**Conclusions:**

The R47H mutation is not sufficient to cause gross phenotypic defects of human pMac under standard culture conditions. However, overlapping transcriptional defects with TREM2 KO supports the hypothesised partial loss-of-function effects of the R47H mutation. Furthermore, transcriptomics can guide us to more subtle phenotypic defects in the R47H cells, such as reduced cell adhesion, and can be used to predict targets for therapeutic intervention.

## Background

Microglia are strongly implicated in the pathogenesis of late-onset Alzheimer’s disease (LOAD). LOAD risk genes, identified by genome-wide association studies (GWAS), include *TREM2*, *PLCG2*, *INPP5D*, and *APOE*, the protein products of which share common signalling pathways in microglia [1, 2]. Furthermore, non-coding LOAD risk variants are largely confined to microglia-specific enhancer regions of the genome [3]. “Disease-associated” microglia have been identified in multiple mouse neurodegenerative disease models, which have a common transcriptional signature that is largely dependent on expression of Triggering Receptor Expressed in Myeloid cells 2 (*TREM2*) [4].

TREM2 is a myeloid cell surface receptor and requires the co-receptor DNAX-Activation Protein 12 (DAP12/TYROBP) for signal transduction. TREM2-DAP12 receptor activation causes activation of spleen-associated tyrosine kinase (SYK). SYK phosphorylates and activates PI3K and PLCγ2, inducing multiple downstream signalling events, resulting in intracellular calcium flux, actin mobilisation, and Akt and ERK activation [5]. TREM2 signalling ultimately regulates many major microglial functions, including phagocytosis [6], chemotaxis [7], survival and proliferation [8, 9], autophagy [10], lipid metabolism [11], and proinflammatory cytokine production [12]. TREM2 has a broad-specificity ligand-binding domain with minor preference for anionic substrates; therefore, it behaves like a scavenger receptor with a long list of putative ligands: various phospholipids and sphingomyelin [13], lipidated apolipoproteins including ApoE [14], bacterial lipopolysaccharides [15], nucleic acids [16], and oligomeric amyloid-β [17].

Nasu-Hakola disease (NHD), a rare genetic disease presenting with early-onset dementia, is linked to mutations in TREM2 that prevent surface expression of mature protein. In contrast, two confirmed Alzheimer’s disease (AD) risk-modifying mutations of *TREM2*, R47H and R62H, occur within the ligand-binding domain of the protein and permit maturation and cell surface expression [18, 19]. R47H TREM2 increases the risk of AD by 2 to 4.5-fold, which makes this rare variant second only to ApoE-ε4 in the strength of association to sporadic AD [18, 20]. The R47H mutation of TREM2 alters the structure of the ligand-binding region and significantly reduces binding of phosphatidylserine (PtdSer), phosphatidylethanolamine, and sphingomyelin in biochemical assays [21, 22]. In cells, reduced intracellular signalling of the R47H mutation was confirmed in response to PtdSer, which is exposed on the surface of apoptotic cells as an “eat-me” signal for professional phagocytes [13, 23]. R47H knock-in mice, crossed with the APPPS1–21 model to simulate AD pathology, suggest that the mutation phenocopies TREM2 knockout or haploinsufficiency models. Namely, there are fewer amyloid plaque-associated microglia, plaques are more diffuse, and microglia exhibit subtle defects in cytokine responses, survival, proliferation, and migration [24]. However, unlike human TREM2, R47H mutation of the mouse gene impairs TREM2 splicing and reduces mRNA and protein expression [25]. This makes the specific effect of the mutation impossible to interpret in R47H knock-in mouse models. Mouse models expressing human *TREM2* should not manifest the same defect. Indeed, humanised R47H TREM2-expressing mice crossed with the 5xFAD model have already been studied and exhibited reduced microglia clustering around amyloid plaques relative to human WT TREM2-5xFAD mice, [26]. The biological mechanisms underlying this phenotype are unexplained.

Given the questions over the validity of different R47H TREM2 mouse models, it is important to explore the cell phenotypes of R47H TREM2 in an authentic human model. Human induced pluripotent stem cells (iPSC) differentiated to microglia-like cells offer the most relevant model to date. Limited study of the phenotypic effects of the R47H mutation has been made in iPSC-derived human microglia-like cells, with conflicting findings regarding the impact of the mutation upon phagocytosis [27, 28]. We have performed a broader and more systematic examination of cell phenotypes using isogenic wildtype, biallelic R47H, and TREM2 KO human iPSC differentiated to iPSC-macrophages (pMac). pMac are a simple model of human microglia that follow a primitive, Myb-independent differentiation, analogous to microglia ontogeny, and display close transcriptional similarity to human foetal microglia [29–31]. We show that TREM2 KO has minimal impact on LPS-induced inflammation; however, deficits are observed in phagocytosis, morphology, migration, and survival. R47H TREM2 has no significant effect on phagocytosis, morphology, migration, or survival, yet reduced IFNγ-primed TLR4-mediated inflammation. However, bulk RNA-seq revealed high overlap of altered gene expression in the R47H TREM2 pMac with the TREM2 KO and suggested that one of the most highly dysregulated pathways was adhesion to extracellular matrix. We used a bioinformatics approach to identify candidate stimuli that were predicted to reverse a subset of transcriptional defects in the TREM2 KO pMac, and tested one of the hits—TGFβ1—to ascertain whether treatment of TREM2 KO and R47H TREM2 pMac would rescue both expression of the vitronectin receptor and adhesion of the cells to vitronectin.

## Methods

### Key materials

Live Cell Imaging solution, Fluo4-AM, CellTracker Deep Red, pHrodo iFL Red STP Ester, NucBlue live cell nuclear stain, NucGreen dead cell nuclear stain, and LIVE/DEAD fixable aqua stain were obtained from Invitrogen. DAPI was from Sigma, and paraformaldehyde (4% in PBS) was obtained from Alfa Aesar. Human annexin V protein was from BD Biosciences, human C5a protein from Peprotech, human TGFβ1 from Miltenyi Biotec, and recombinant human IFNγ and recombinant truncated human vitronectin from Gibco. Cytochalasin D was from Cayman Chemicals, Bafilomycin A1 from Abcam, and Jasplakinolide from Santa Cruz. PSB0739, MRS2179, MRS2211, and cilengitide were all obtained from Tocris. ADP, BSA, and crystal violet were from Sigma.

### iPSC culture

iPSC lines BIONi010-C (control, BioSample ID: SAMEA3158050, ECACC ID: 66540023), BIONi010-C-7 (R47H TREM2, BioSample ID: SAMEA4454010, ECACC ID: 66540369), and BIONi010-C-17 (TREM2 KO, BioSample ID: SAMEA104386270, ECACC ID: 66540632) were obtained from Bioneer and are available from the European Collection of Authenticated Cell Cultures (ECACC). The parent line BIONi010-C was re-programmed by Bioneer with a non-integrating episomal vector, using normal adult human skin fibroblasts sourced from Lonza (#CC-2511). For some supplementary data, pMac differentiated from SFC840-03-03 iPSC were used, a previously published line derived from dermal fibroblasts from a disease-free donor recruited through the Oxford Parkinson’s Disease Centre [32]. iPSC were cultured in mTeSR™1 media (STEMCELL Technologies), on hESC-qualified Geltrex-coated plates (Gibco), passaging as clumps using 0.5 mM EDTA in PBS. Large-scale SNP quality-controlled batches were frozen at p15–25 and used for experiments within a minimal number of passages post-thaw to ensure consistency. An Illumina Omniexpress 24 v1.2 SNP microarray analysis was performed to verify genomic integrity, as previously described in [30].

### Human iPSC differentiation to pMac

iPSC were differentiated to primitive, tissue-type macrophages as previously described [29]. In brief, 4 × 10^6^ iPSC were seeded into an Aggrewell-800 plate well (STEMCELL Technologies) to form embryoid bodies, in mTeSR™1 media (STEMCELL Technologies), and fed daily with medium plus growth factors: 50 ng/mL BMP4 (Peprotech), 50 ng/mL VEGF (Peprotech), and 20 ng/mL SCF (Miltenyi Biotec). In a modification to the previously published protocol, the embryoid bodies were cultured for 5–6 days in growth factors instead of 4 days, and after the first 2 days they were transferred into low-adherence 6-well plates. Embryoid bodies were then differentiated in T175 flasks (150 per flask), known as “differentiation factories”. iPSC-macrophage precursors (pMacpre), emerging into the supernatant after approximately 2–3 weeks, were harvested weekly, were plated in their final assay format, and were differentiated to pMac for 6–10 days at 37 °C/5% CO_2_, in X-VIVO15 with 100 ng/mL M-CSF, 2 mM Glutamax, 100 U/mL penicillin, and 100 μg/mL streptomycin.

### DNA sequencing of R47H mutation

DNA was extracted from a cell pellet of iPSC using the DNeasy Blood & Tissue kit from QIAGEN. A PCR reaction in 25 μL was performed using Phusion HF buffer, 0.5 units Phusion HF DNA polymerase, 200 μM dNTPs, 500 nM forward primer (AAACACATGCTGTGCCATCC), 500 nM reverse primer (CACAGACGCCCAAAACATGAG), and genomic DNA (50–100 ng). PCR reaction: 1 × (98 °C, 30 s), 30 × (98 °C, 5 s; 60.7 °C, 10 s; 72 °C, 15 s), 1 × 72 °C, 5 min. PCR products were sent for sequencing (Eurofins) with the reverse primer: TGATGGCTGTGCTCCCATTC.

### Western blotting

pMacpre were seeded in either 12-well plates (8 × 10^5^ pMac per well) or 6-well plates (1.5 × 10^6^ pMac per well) and differentiated for a week in macrophage media. Stimulations with a TREM2-activating antibody (goat polyclonal antibody against human TREM2 AF1828 from R&D Systems) used a concentration of 2.4 μg/1 × 10^6^ cells (3.84 μg/mL) for 10 min. Stimulations with dead SH-SY5Ys used a ratio of 3:1 SH-SY5Ys to pMac, with an initial 1 h incubation at 4 °C to allow cells to settle, followed by incubation at 37 °C for the time periods indicated. After washing with PBS, pMac were lysed directly in modified RIPA buffer (50 mM Tris-HCl (pH 7.4), 1% Triton X-100, 0.5% sodium deoxycholate, 0.1% sodium dodecyl sulphate, 150 mM sodium chloride, 2 mM ethylenediaminetetraacetic acid, 50 mM sodium fluoride) with protease and phosphatase inhibitor cocktails (Roche). Lysates were sonicated for 30 s at medium power and centrifuged at 14000×*g* for 3 min to a pellet-insoluble material. Triton X-100-soluble proteins were boiled with 1× LDS sample buffer with reducing agent (Thermo Fisher), and samples were resolved by denaturing SDS-PAGE with 8–16% Tris-glycine gels (Thermo Fisher). Subsequently, the separated proteins were transferred to low-fluorescence 0.2 μM-pore PVDF membrane (Thermo Fisher) using a Pierce Power Blotter semi-dry transfer apparatus (Thermo Fisher). Membranes were blocked for 0.5–2 h with iBind FD solution (Thermo Fisher) and incubated with primary antibodies in iBind solution overnight at 4 °C. Primary antibodies for TREM2, phospho-SYK (Y525/Y526), total SYK, and GAPDH were purchased from commercial sources and are listed in Table 1. Membranes were washed 3 × 10 min with Tris-buffered saline and 0.05% Tween-20 (TBS-T) and probed for 1 h at room temperature (RT) with secondary antibodies in iBind FD solution. Membranes were washed 4–6 × 10 min with TBS-T and developed with an Odyssey imaging system (LI-COR®). Optical densities of immunoreactivity were quantified using ImageStudio™ Lite 5.2 software and normalised to the GAPDH control.

**Table 1:** Antibodies

Target	Species raised in	Antibody conjugate	Identifier	Supplier	Conc.	Use
TREM2	Goat	–	AF1828	R&D Systems	3.84 μg/mL / 1:30	Stimulus/ICC
TREM2	Rabbit	–	EPR20243	Abcam	1:500	WB
SYK pY525/ pY526	Rabbit	–	MA5-14918	Thermo Fisher	1:200	WB
Total SYK	Mouse	–	4D10	CST	1:500	WB
Fibronectin	Rabbit	–	15613-1-AP	Proteintech	1:500	WB
GAPDH	Rabbit	–	G9545	Sigma	1:2000	WB
Tubulin	Mouse	–	T5168	Sigma	1:1000	WB
Rabbit IgG	Goat	IRDye® 800CW	926-32211	LI-COR	1:3000–1:5000	WB
Mouse IgG	Donkey	IRDye® 680RD	926-68072	LI-COR	1:3000–1:5000	WB
Calnexin	Rabbit	–	ab22595	Abcam	1:1500	ICC
TGN-46	Rabbit	–	ab50595	Abcam	1:100	ICC
LAMP1	Rabbit	–	9091	CST	1:200	ICC
RAB9	Rabbit	–	5118	CST	1:100	ICC
TUJ1	Mouse	–	801201	Biolegend	1:500	ICC
Goat IgG	Donkey	Alexa Fluor® 488	A11055	Thermo Fisher	1:1000	ICC
Rabbit IgG	Donkey	Alexa Fluor® 568	A10042	Thermo Fisher	1:500	ICC
Rabbit IgG	Donkey	Alexa Fluor® 647	A31573	Thermo Fisher	1:500	ICC
Mouse IgG	Donkey	Alexa Fluor® 647	A31571	Thermo Fisher	1:500	ICC
IL-6	Rat	–	14-7069-81	Thermo Fisher	4 μg/ml	ELISA – capture
IL-6	Rat	Biotinylated	13-7068-81	Thermo Fisher	2 μg/ml	ELISA – detection
sTREM2	Rabbit	–	ab209814	Abcam	8 μg/ml	ELISA – capture
sTREM2	Goat	Biotinylated	BAF1828	R&D Systems	1.5 μg/ml	ELISA – detection
CD11B	Mouse (IgG1κ)	APC	301309	Biolegend	1:25	FC
CD14	Mouse (IgG1)	PE	21620144	Immuno-tools	1:25	FC
CD45	Mouse (IgG1)	APC	21335014	Immuno-tools	1:25	FC
αVβ3	Mouse (IgG1κ)	APC	304415	Biolegend	1:50	FC
αVβ5	Mouse (IgG1)	PE	FAB2528P	R&D Systems	1:50	FC
Control	Mouse (IgG1κ)	APC	400119	Biolegend	1:50	FC
Control	Mouse (IgG1)	PE	21335014	Immuno-tools	1:25	FC
Control	Mouse (IgG1)	APC	21275516	Immuno-tools	1:25	FC
Control	Mouse (IgG1)	PE	IC002P	R&D Systems	1:50	FC

List of antibodies used in this study. *WB* Western blot, *ICC* immunocytochemistry, *FC* flow cytometry

### Flow cytometry for surface proteins

pMac were lifted from 6-well plates by incubation with StemPro Accutase (Gibco) for 10 min at 37 °C. The cells were washed with PBS and blocked in FACS buffer (PBS, 1% FCS, 10 μg/mL human IgG) for 10 min at RT. 2 × 10^5^ cells per sample were stained with directly conjugated primary antibodies against CD11b, CD14, CD45, αVβ3, or αVβ5, for 30 min on ice. A viability stain was included with the αVβ3 and αVβ5 antibodies (LIVE/DEAD fixable aqua, Invitrogen). Cells were then washed 2–3 times with FACS buffer and fixed with 4% paraformaldehyde in PBS (Alfa Aesar) for 10 min at RT. Cells were spun down and re-suspended in PBS. CD11b, CD14, and CD45 staining was analysed with a FACSCalibur flow cytometer (BD Biosciences), whereas αVβ3 and αVβ5 were analysed with an LSRFortessa X20 flow cytometer (BD Biosciences). Fluorophore-conjugated isotype controls from the same manufacturers were used. The antibodies are listed in Table 1.

### Cell surface protein purification

pMacpre were seeded at 2 × 10^6^ cells/well in 6-well plates and differentiated for a week. Cell surface proteins were extracted using a Pierce Cell Surface Protein Isolation kit (Thermo Fisher). In brief, the plates of pMac were placed on ice and washed twice with cold PBS with Ca^2+^ and Mg^2+^. Cell surface proteins were biotinylated with 0.5 mg/well of sulfo-NHS-SS-biotin for 30 min on ice. Free biotin was quenched with Quenching Buffer (Thermo Fisher); the cells were gently scraped into tubes, spun down, and washed with Tris-buffered saline. The cell pellets were lysed in RIPA buffer (50 mM Tris-HCl (pH 7.4), 1% Triton X-100, 0.5% sodium deoxycholate, 0.1% sodium dodecyl sulphate, 150 mM sodium chloride, 2 mM EDTA, 50 mM sodium fluoride) and briefly sonicated, and protein content was measured by Bradford protein assay (Bio-Rad). Total protein concentrations between samples were normalised, and an aliquot of normalised whole-cell lysate was saved for Western blotting. The remaining homogenates were incubated with twice the volume of Neutravidin beads (Thermo Fisher) overnight at 4 °C with rotation. Beads were washed three times by centrifugation and re-suspension in Wash Buffer (Thermo Fisher). Cell surface proteins were eluted from the beads by boiling for 15 min with 1× “LDS Sample buffer” (Thermo Fisher) with 50 mM DTT. Cell surface proteins and whole-cell lysates (containing proteins soluble in 1% Triton X-100) were analysed by Western blotting, including a negative control of non-biotinylated cells.

### Immunocytochemistry

pMacpre were seeded at 4 × 10^4^ cell/well in optically clear bottom CellCarrier 96-well plates (Perkin Elmer) and differentiated in macrophage media for a week. For cell surface staining of live pMac: cells were washed with cold PBS and incubated on ice for 20 min with primary antibody in a live-cell blocking buffer (PBS with 5% BSA and 10 μg/mL human IgG). Cells were washed three times for 5 min with cold PBS, on ice, and incubated with secondary antibody in live-cell blocking buffer on ice for 15 min. After three washes with cold PBS, cells were fixed with 2% paraformaldehyde in PBS for 20 min at RT, then washed with PBS. The cells were permeabilized and counterstained with DAPI as below. For total staining of permeabilized cells: cells were fixed in 2% paraformaldehyde in PBS (Alfa Aesar) for 20 min at RT, washed with PBS, permeabilized with 0.1% Triton X-100 in PBS for 10 min at RT, and blocked overnight at 4 °C in blocking buffer (PBS, 0.05% Triton-X100, 10% normal donkey serum, 5% BSA, 0.01% NaN_3_). Cells were incubated with primary antibodies for 1 h at RT, washed 3 × 15 min with 0.3% Triton X-100 in PBS, and incubated with secondary antibodies for 1 h at RT. Primary antibody for TREM2 (AF1828, 1:30, R&D Systems) was used in combination with either of the following primary antibodies for subcellular markers: TGN-46, calnexin, RAB11, LAMP1, TUJ1, and RAB9. Secondary antibodies were anti-goat IgG-Alexa Fluor 488 for TREM2, anti-rabbit IgG-Alexa Fluor 568 for TGN-46, calnexin, RAB11 and LAMP1, anti-mouse IgG-Alexa Fluor 647 for TUJ1, and anti-rabbit IgG-Alexa Fluor 647 for RAB9. The antibodies used are listed in Table 1. A DAPI nuclear counterstain (1:2000, Sigma) was incubated for 15 min at RT, and the cells washed 4 × 15 min. Images were acquired with an Opera Phenix High Content Screen System (Perkin Elmer) with a 63x water objective, Z-stacks of 9 fields per well, and 3 wells per condition. Columbus 2.7 software (Perkin Elmer) was used to generate a co-localization index for co-localization of TREM2 with intracellular markers. The analysis pipeline defined intracellular regions as “ER”, or “TGN”, or “lysosome” based upon the intensity and pattern of subcellular marker staining: “ER” is a tubular pattern of calnexin, “TGN” is a single large focal structure of TGN46 per cell, and “lysosome” is a punctate pattern of LAMP1. Calculated within these defined regions, the co-localization index used is (intensity of TREM2)/ (intensity of subcellular marker).

### ELISAs

For TGFβ1, pMacpre were seeded at 1.2 × 10^6^ cell/well in 6-well plates in full macrophage media, and the media were replaced after 7 days with 1 mL full media ± 2 μM OXSI-2 for a further 24 h incubation at 37 °C/ 5% CO_2_. Supernatants were spun down and stored in aliquots at − 80 °C. TGFβ1 was quantified using a Human/Mouse TGF beta-1 Uncoated ELISA Kit (Invitrogen), in accordance with the manufacturer’s protocol.

For sTREM2/IL-6/TNF, pMacpre were seeded at 4 × 10^4^ cells/well in optically clear bottom CellCarrier 96-well plates (PE) and differentiated in macrophage media for a week. In triplicate wells, cells were stimulated for 24 h ± 100 ng/mL IFNγ. All wells were given a full media change to 70 μL macrophage media ± *E. coli* lipopolysaccharide (LPS) and incubated for 4 h at 37 °C/ 5% CO_2_. Supernatants were spun down and stored in aliquots at − 80 °C. For cell counting, cells were stained with NucBlue Live ReadyProbes Reagent (Invitrogen), and nuclei counting was performed using an EVOS FL Auto automated microscope (Thermo Fisher) at 4x objective, 4 fields per well, and CellProfiler 2.2 software [33]. Supernatant TNF was quantified using a TNFα Human Uncoated ELISA Kit (Invitrogen), in accordance with the manufacturer’s protocol. An in-house ELISA was used to measure IL-6 and sTREM2, from the same supernatants measured for TNF, except with triplicate wells pooled. For in-house IL-6/TREM2 ELISA procedure, Greiner high-bind 96-well plates (Sigma) were coated with appropriate capture antibody (detailed in Table 1 for each ELISA) overnight at 4 °C. Plates were washed with PBS + 0.05% Tween20 and incubated with blocking buffer (PBS, 0.05% Tween20, and 1% BSA) to block non-specific binding sites. A standard curve was generated using human recombinant IL-6 or TREM2 (Sino Biologicals). Standard and diluted supernatants were incubated for 2 h at RT. After washing, plates were incubated with the appropriate biotinylated detection antibody (Table 1) for 1 h at RT. Plates were washed 3x in PBS-T, then incubated with HRP-conjugated streptavidin (Thermo Fisher Scientific) for 1 h at RT. Plates were washed and incubated with 1-Step Ultra TMB ELISA substrate solution (Thermo Fisher Scientific). The reaction was stopped with 2 N H_2_SO_4_, and the chemiluminescent signal was measured on a plate reader at 450 nm. Data from each well was normalised to the average cell count for that condition, and further normalised to the average pg/mL/cell for the whole ELISA plate. The in-house ELISA antibodies are listed in Table 1.

### Calcium assay

pMacpre were seeded at 1 × 10^4^ cells/well in optically clear bottom CellCarrier 384-well plates (Perkin Elmer) and differentiated in macrophage medium for 7 days. The stimuli used were 0.5 mM ATP (Sigma) and 10 μg/mL TREM2 antibody (R&D Systems). A 384-well plate containing stimuli was prepared for transfer onto the pMac. pMac were loaded with 25 μL of 4 μM calcium-sensitive dye Fluo4-AM (Thermo Fisher Scientific) in the presence of 0.5% pluronic acid (Life technologies) diluted in HBTS buffer (HEPES Buffered Tyrode’s Solution: NaCl 135 mM, KCl 5 mM, MgCl_2_ 1.2 mM. CaCl_2_ 2.5 mM, HEPES 10 mM, glucose 11 mM, pH 7.2) for 1 h at RT. pMac were washed with HBTS before the plates of pMac, and stimuli were loaded onto the FLIPR Tetra (Molecular Devices), a high-throughput cell-based screening system with a robotic pipettor. Each condition was run in quadruplicate. Relative fluorescent units (RFU) of the assay plate were read with the excitation/emission pairs 470–495 nm LEDs and 515–575 nm emission filters. Settings were adjusted in order to have values of ~ 1000 RFUs at baseline. Basal fluorescence was measured for 1 min, and following injection of stimuli, the response was recorded for 5 min at reading intervals of 5 s using the ScreenWorks software. Data was exported as maximum-minimum signal and RFU normalised to baseline values set to 100%.

### Generation of dead SH-SY5Ys

SH-SY5Ys (ATCC) were cultured in T75 flasks with DMEM/F12 media (Gibco) with 10% FBS (Sigma) and penicillin/streptomycin (Invitrogen), and maintained at 37 °C/5% CO_2_. Cells were harvested with TrypLE Express (Gibco), washed with Hank’s Balanced Salt Solution (HBSS, Gibco), centrifuged at 400×*g* for 5 min, and re-suspended in 2 mL Live Cell Imaging Solution (LCIS, Invitrogen). Paraformaldehyde (Alfa Aesar) was added to a final concentration of 2%, and the cells were fixed for 10 min at RT. The cells were washed again with HBSS and centrifuged at 1200×*g* for 7 min.

### Generation of rat cortical synaptosomes

Two wildtype female ex-breeder Sprague-Dawley rats (Charles River) were sacrificed using a CO_2_ procedure, in accordance with the approved humane killing protocols detailed in Schedule 1 of the Animals in Scientific Procedures Act, 1986, and the brain cortices dissected. Synaptosomes were purified from the fresh cortices using a previously described method of Percoll gradient fractionation, with four Percoll gradients per rat [34]. An aliquot of the purified synaptosomes was dissolved in 1% NP-40 and the protein concentration determined by Bradford assay. Accordingly, the synaptosomes were diluted to 1 mg/mL of their total protein content with HEPES-buffered media (pH 7.4, 140 mM NaCl (VWR), 5 mM KCl (VWR), 5 mM NaHCO_3_ (Sigma), 1.2 mM NaH_2_PO_4_ (Sigma), 1 mM MgCl_2_.6H_2_O (Sigma), 10 mM glucose (VWR), 1 mg/ml BSA (Sigma), 10 mM HEPES (Sigma)) with 5% (v/v) DMSO and frozen in single-use aliquots at − 80 °C. Thawed synaptosomes were characterised by negative staining transmission electron microscopy, performed by the Sir William Dunn School of Pathology Electron Microscopy Facility. Upon thawing, synaptosomes were centrifuged at 3000×*g* for 10 min at 4 °C and washed once with Live Cell Imaging Solution (Invitrogen), to remove residual BSA before pHrodo-labelling.

### Annexin V-FITC staining for phosphatidylserine

Phosphatidylserine exposure of phagocytic cargo was visualised using an annexin V-FITC Apoptosis Detection Kit (Abcam). One day prior to staining, SH-SY5Ys were seeded to 50% confluence in a 24-well plate. Synaptosomes were thawed, washed, and re-suspended in annexin binding buffer, and approximately 0.3 μg per well was added to empty wells of the plate, allowing an hour to settle at 37 °C/5% CO_2_. SH-SY5Ys were washed with HBSS and some wells fixed with 2% paraformaldehyde for 10 min, before another wash, replacing with annexin binding buffer containing NucBlue. Both SH-SY5Ys and synaptosomes, except for unstained controls, were stained with 1:70 annexin V-FITC and 1:70 propidium iodide for 5 min. Propidium iodide stains nuclei of permeable cells, controlling for annexin V staining of the plasma membrane inner leaflet. The plate was imaged at 37 °C/5% CO_2_ using an EVOS FL Auto automated microscope (Thermo Fisher) with on-stage incubator at 40x objective with phase and using the DAPI, GFP, and Texas Red light cubes.

### Phagocytosis assays

pMacpre were seeded at 2 × 10^4^ cells/well in optically clear bottom CellCarrier 96-well plates (Perkin Elmer) and differentiated in macrophage media for a week. Cells were stained for 45 min at 37 °C/5% CO_2_ with 1 μM CellTracker Deep Red (Invitrogen) and 1 drop/mL NucBlue Live ReadyProbes Reagent (Invitrogen). Cells were washed with PBS, and then incubated for 1 h at 37 °C/5% CO_2_ with 100 μL of Live Cell Imaging Solution (LCIS, Invitrogen) ± phagocytosis inhibitors, before addition of phagocytic cargo. Phagocytosis inhibitors used for validation were 10 μM cytochalasin D (Cayman), 1 μM bafilomycin A1 (Abcam), 1 μM jasplakinolide (Santa Cruz), and 2 μg unlabelled human recombinant annexin V (BD Biosciences). Annexin V was added to well immediately prior to addition of phagocytic cargo. The phagocytic cargo- synaptosomes or dead SH-SY5Ys- were stained with pHrodo iFL Red STP Ester (Invitrogen), using 20 μg of dye per 1 mg synaptosomes, or 12.5 μg of dye per 1 × 10^6^ SH-SY5Ys, aiming for a final concentration of 40 μg/mL. pHrodo-labelling was performed for 30 min at RT, protected from the light, in a low protein-binding tube. Cargo was washed twice with HBSS, (centrifugation: 3000×*g* synaptosomes, 1200×*g* dead SH-SY5Ys) and re-suspended in LCIS to a concentration of 0.6 μg/μL synaptosomes or 8 × 10^5^ cells/mL SH-SY5Ys, and 50 μL/well added to the pMac. Phagocytosis was performed at 37 °C/5% CO_2_ for 0.5–5 h, in triplicate wells. Cells were fixed with 2% paraformaldehyde in PBS (Alfa Aesar) for 15 min at RT and washed with PBS before imaging. For staining TREM2, TUJ1, and RAB9a, immunocytochemistry was performed on permeabilized cells as described above. Images were taken with an INCell Analyzer 6000 high-content imaging system (GE Healthcare Life Sciences) with a 40x objective, 9 fields/well on a single plane. Images were quantified with Columbus 2.7 software (Perkin Elmer). The parameters measured for each field were average number of spots/cell, the sum of the spot areas, and the % spot-positive cells. Data was averaged for the technical replicates and normalised to the overall plate average, to adjust for differences between plates.

### Transwell assays

pMacpre were seeded at 1.2 × 10^6^ cells/well in 6-well plates and differentiated in macrophage media for a week. Cells were dissociated with StemPro Accutase (Gibco) for 10 min at 37 °C and washed with PBS. pMac were re-suspended in cold macrophage media, and 100 μL with 5.5 × 10^4^ cells was pipetted onto transwells (PET with 5 μm pores, Sarstedt) suspended over wells of an empty 24-well plate. In total, 600 μL of macrophage media ± inhibitors was added beneath the transwells and incubated 30 min at RT, before the chemotactic stimulus was added: 30 μM ADP (Sigma) or 3 nM human recombinant C5a (Peprotech). The 24-well plates were incubated for 6 h to allow cell migration. After cell migration, the transwells were gently rinsed with PBS, transferred to a fresh 24-well plate, and fixed with 4% paraformaldehyde (Alfa Aesar) for 20 min at RT. Cells were stained with NucBlue (Invitrogen) nuclear stain and imaged with an EVOS FL Auto automated microscope (Thermo Fisher), 4× with DAPI light cube, set up to scan each transwell in full and generate a knitted single image. The transwells were swabbed with a cotton wool bud to remove cells on the top surface, leaving behind only migrated cells, transferred into a fresh plate with fresh PBS, and imaged again with the same settings. Nuclei counting was performed with CellProfiler2.2 software [33], and for each transwell, the % migration was calculated: (no. cells in second scan) ÷ (no. cells in first scan) × 100. Treatments were performed in duplicate and duplicate wells were averaged, then normalised to the average % migration for the entire plate, to control for age-dependent differences in cell speed.

### Survival assay

pMacpre were seeded at 4 × 10^4^ cells/well in three optically clear bottom black 96-well plates (Costar) and differentiated in macrophage media. After 7 days, a full media change was performed to 100 μL macrophage media ± M-CSF, triplicate wells for each condition on each plate. Plates were incubated at 37 °C/ 5% CO_2_ for 3, 7, or 10 days, and the 10-day plate received a 50% media change at 7 days. At the end of each incubation, cells were stained 20 min (37 °C/5% CO_2_) with the ReadyProbes Cell Viability Imaging Kit (Invitrogen). Nuclei counting was performed using an EVOS FL Auto automated microscope (Thermo Fisher), 4x objective with DAPI and GFP light cubes, 4 fields/well, and CellProfiler 2.2 software [33]. Data was presented as (mean number of dead cells/mean number of total cells) × 100 for each condition.

### RNA-seq sample and library preparation

pMacpre were seeded at 1.5 × 10^6^ cells/well in 6-well plates at 7, 8, and 9 weeks after setting up differentiation factories, and they were differentiated in macrophage media for 7 days. Cells were washed once with PBS, aspirated thoroughly, and lysed by addition of 350 μL Buffer RLT (QIAGEN) with 1% (v/v) 2-mercaptoethanol. Plates were stored at − 80 °C until all samples were collected, and then lysates were passed through QIAshredder columns (QIAGEN) and the total RNA extracted using a QIAGEN RNeasy Mini kit, according to the manufacturer’s protocol using on-column DNase treatment (QIAGEN). RNA samples were eluted in 30 μL of RNase-free water. The quantity and quality of RNA was measured by Nanodrop and RNA Tapestation (Agilent), with measured RNA integrity (RIN) values ≥ 8.4. Poly-A library preparation and sequencing (HiSeq 4000, 75 bp paired-end reads), and basic data processing, was conducted at the Oxford Genomics Centre, Wellcome Centre for Human Genetics.

### Analysis of RNA-seq data

Quantified transcript abundance counts were obtained with the tool Salmon (v0.12.0)(PMID: 28263959) using mapping-based mode with default parameters, automatic library type-inference, and the additional command line arguments “validatemappings”, “seqBias”, “posBias”, and “gcBias”, in order to enable selective alignment of the sequencing reads, and account for sequence-specific biases, fragment-level GC biases, and 5′ or 3′ positional biases in the data. The paired-end sequencing reads for each sample were mapped to the human reference transcriptome (GRCh38; Ensembl release 95), which combined cDNA and ncRNA. The transcript abundances were imported and summarised to gene-level counts using the R library “tximport” (PMID: 26925227)**.**

Only the protein-coding genes were used, and data were filtered to include genes (*n* = 15,341) with > 10 counts across all samples. Differential gene expression between the TREM2 KO vs WT and TREM2 R47H vs WT was performed on genes expressed across all samples with the R library “DESeq2” (PMID: 25516281) using the Wald test, and LFC shrinkage was performed using the “ashr” method (PMID: 27756721). We considered a gene to be differentially expressed at FDR < 0.05. Principal component analysis was performed using the “prcomp” function in R, on variance-stabilising transformed data for all genes. PC1 was added as a covariate to the design model, to remove the influence of the covariate “differentiation age” on the differential expression. Gene ontology enrichment analysis was performed with the R library ClusterProfiler (PMID: 22455463) and adjusted *p* values for multiple testing following a Benjamini-Hochberg correction, and terms were filtered out if they were associated with fewer than 10 differentially expressed genes. All plotting was performed with multiple libraries in R.

### Gene functional network and clustering method

A combined protein-protein interaction (PPI) network was created based on diverse resources: BioGRID 3.4 (accessed on September 2017) [35], HitPredict (accessed on September 2017) [36], IntAct (accessed on September 2017) [37], STRING (accessed on September 2017, restricted to *Homo sapiens* and experimental scores higher than zero) [38], CORUM (accessed on September 2017) [39], and Reactome (accessed on September 2017) [40]. The combined network consisted of a total of 20,591 genes and 1,973,967 interactions. A gene functional network was built for the differentially expressed genes between TREM2 KO and WT samples, by using the top 500 K PPI interactions. To identify modules of highly interconnected genes in the network, we employed “cluster_louvain” function in “igraph” R library [41], where proteins in clusters with fewer than 30 members were grouped into a cluster with the label “0”.

### Randomisation for PPI network analysis

The randomisation approach takes into account both coding sequence length and the number of PPIs of the corresponding proteins. For every input gene, a random gene with similar coding sequence length and similar connectivity of its corresponding protein was selected, by applying a binning approach (PMID: 25319962). A total of 10,000 random gene sets, of the same size as the number of differentially expressed genes, were tested for their connectivity within the combined PPI network, and the number of connections were compared to the one obtained using the list of differentially expressed genes for TREM2 KO vs WT samples. The PPI analysis was performed on the basis of pairs of genes (using ensembl gene IDs annotated in combined PPI network), which means that an interaction between two genes is reported and counted if any of the possible gene products show an interaction.

### External dataset comparison

Processed FPKM counts for RNA-seq data from Abud et al. [42] were downloaded from GEO: GSE89189 and were filtered to include only protein-coding genes that were common with the RNA-seq data in this study. The study batch-effect of the combined log2-transformed FPKM gene expression levels was removed by using the “ComBat” function in the “sva” R library (PMID: 22257669) and hierarchical clustering of the Euclidean distances of the samples was performed using the “ward. D2” method, and the dendrogram visualised with the “dendextend” R library. A scatter plot was made using R library “ggplot2” of significant (FDR < 0.05) differentially expressed genes for TREM2 KO vs WT from the Claes et al. RNA-seq study [27], against the significant results for TREM2 KO vs WT from the current study.

### Ingenuity pathway analysis

For each of the PPI modules of differentially expressed genes, we identified upstream regulators using Ingenuity Pathway Analysis software [43]. The algorithm takes into account the observed gene expression changes, and the manually curated Ingenuity Knowledge Base, to infer the “activated” or “inhibited” state of upstream regulators (including transcription factors, small molecules, microRNA, and other genes). The “Regulators Effect” algorithm takes into account each identified upstream regulator and links it through the Ingenuity Knowledge Base to functions, phenotypes, and disease. Briefly, it takes the best matching pair (regulator and downstream effect networks) and tests for overlapping sets with a Fisher exact test and then iteratively groups regulators to both increase consistency and reduce redundancy of the network.

### qRT-PCR

pMacpre were seeded at 0.8 × 10^6^ cells/well in 12-well plates, differentiated in macrophage media for 7 days, and stimulated ± 50 ng/mL TGFβ1 (Miltenyi Biotec) for 24 h. Cells were washed once with PBS, aspirated thoroughly, and lysed by addition of 350 μL Buffer RLT (QIAGEN) with 1% (v/v) 2-mercaptoethanol. Plates were stored at − 80 °C, and RNA extracted as detailed for the RNA-seq sample preparation above. Reverse transcription was performed using a High-Capacity RNA-to-cDNA kit (Applied Biosystems), with 400 ng RNA input per reaction, following the manufacturer’s protocol. qRT-PCR was performed using TaqMan probes and TaqMan Gene Expression Master-mix (Applied Biosystems) in a 384-well PCR plate, 2 μL cDNA in a final volume of 6 μL per well, on a QuantStudio 5 qRT-PCR machine (Applied Biosystems). Taqman probes were *CHCHD2* (Hs00855326_g1), *ITGAV* (Hs00233808_m1), *ITGB3* (Hs01001469_m1), *ITGB5* (Hs00174435_m1), *FN1* (Hs01549976_m1), *LAMB2* (Hs00158642_m1), *SDC4* (Hs01120908_m1), *GPC4* (Hs00155059_m1), and *TBP* (Hs00427620_m1). Samples were run in triplicate wells. ΔΔCt values were calculated using the average Ct for each triplicate: ΔCt was generated by subtraction of the average Ct for reference gene *TBP*, and then the ΔCt was normalised to the average ΔCt for all WT unstimulated samples (by subtraction).

### Adhesion assay

pMacpre were seeded at 1.2 × 10^6^ cell/well in 6-well plates in full macrophage media, and the media replaced after 6 days with 1 mL full media ± 50 ng/mL TGFβ1 (Miltenyi Biotec) for a further 24 h incubation at 37 °C/ 5% CO_2_. Wells of a clear 96-well flat-bottomed plate were coated with 0.5 μg/well (1.56 μg/cm^2^) truncated vitronectin (Gibco) in PBS, by incubation at room temperature for 1 h. The vitronectin was aspirated, and remaining non-specific binding sites were blocked with a solution of 10 mg/mL denatured bovine serum albumin (BSA) in PBS for 1 h. Wells were washed once with PBS, and 50 μL of Live Cell Imaging Solution (LCIS; Invitrogen) ± cilengitide added (10 μM final well concentration). pMac dissociated from the 6-well plates by StemPro Accutase (Gibco) were pelleted and re-suspended in LCIS, and 50 μL added to the vitronectin-coated wells at a density of 5 × 10^4^ cells/well. Cells were also added to no-vitronectin BSA-blocked wells as a control for non-specific binding. Adhesion was performed for 3 h at 37 °C/5% CO_2_, and then the plate was washed once with PBS and cells fixed for 10 min in 4% paraformaldehyde at RT. Following two PBS washes, the cells were stained with crystal violet solution (0.1% (w/v) in 50 mM MES, 150 mM NaCl, pH 6) for 1 h at RT. Wells were washed 3 times with ddH_2_O, and the cells and dye were solubilised with 50 μL of 10% (v/v) acetic acid. Absorbance was measured at 570 nm on a microplate spectrometer, normalised to the non-specific binding controls.

### Statistical analysis

Statistical analysis of the data was performed in GraphPad Prism software (version 7 for Windows), GraphPad Software, La Jolla, CA, USA, www.graphpad.com. All “n” numbers represent independent biological replicates, i.e., separate harvests of the differentiation cultures, with experiments performed independently on different weeks. Means were obtained from three or more independent repeats, and paired *t*-tests and one-way or two-way ANOVAs performed where appropriate, with Bonferroni, Sidak, or Dunnett corrections for multiple comparison. *P* values < 0.05 were considered to be significant and are indicated as follows: *p < 0.05, **p < 0.01, *** *p* < 0.001, **** *p* < 0.0001.

## Results

### R47H TREM2 has reduced cell surface expression compared with WT

Human microglia can be effectively modelled in vitro by the differentiation of human iPSC, via our previously published protocol for primitive, tissue-type macrophages, pMac [29, 31]. pMac have a similar transcriptional signature to iPS-microglia co-cultured with neurons and express high levels of microglial genes [30]. We used three iPSC lines generated by Bioneer with the R47H *TREM2* mutation inserted homozygously, and a *TREM2* knockout, both on the same genetic background as the control WT iPSC line. We independently validated the R47H mutation by DNA sequencing and the TREM2 knockout by Western blotting (Additional file 1: Figs. S1, S3). After differentiating the iPSC lines to pMac, we confirmed that a similarly high efficiency of differentiation was achieved between the three lines, using surface markers CD11b, CD14, and CD45 (Additional file 1: Fig. S3). Interestingly, although > 80% of cells were positive for CD11b/CD14/CD45 in all three genotypes, we found that both the R47H TREM2 and TREM2 KO lines had significantly lower CD11b expression and higher CD14 expression than the WT, perhaps signifying changes to basal activation state.

**Fig. 1:**
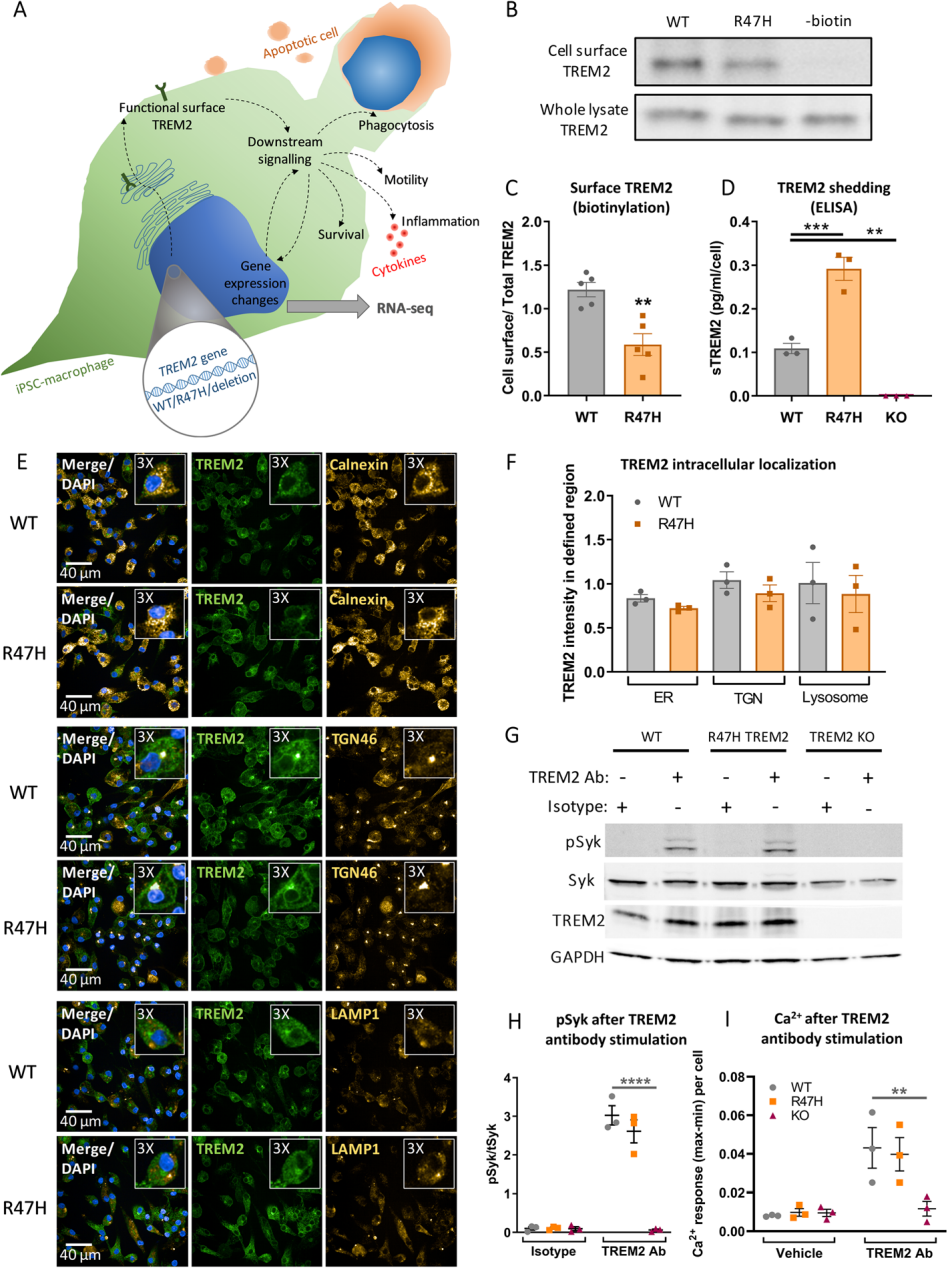
Reduced cell surface localization of R47H TREM2 does not impair antibody-mediated activation of TREM2 in pMac. **a** Schematic of microglia phenotypes investigated in this study. **b**, **c** Reduced cell surface expression of R47H TREM2. Cell surface proteins on pMac were biotinylated and pulled down, the level of TREM2 protein enrichment was measured by Western blotting vs whole cell lysate, probed on separate blots. **c** Means ± SEM, for *N* = 5 harvests measured on separate Western blots. 2-tailed paired t-test: ** *p* = 0.0011 for R47H versus WT. **d** Increased sTREM2 production from R47H TREM2 pMac. sTREM2 were measured from unstimulated pMac supernatants by ELISA. Means ± SEM, for *N* = 3 harvests measured on same ELISA plate, data for each harvest was normalised to the average cell count. 1-way ANOVA, with Dunnett’s post hoc test: *p* = 0.0005 R47H versus WT (***), *p* = 0.0066 KO versus WT (**). **e**, **f** Co-localization of TREM2 with subcellular compartment markers in fixed and permeabilized pMac, images are a confocal slice at 4 μm, taken with Opera Phenix microscope. Inset panels are × 3 magnification of a selected cell. Calnexin used as a marker for ER, TGN46 for TGN, and LAMP1 for lysosomes. **f** Co-localization expressed as a ratio of TREM2 intensity to compartment marker intensity, in regions automatically segmented by high marker staining. Means ± SEM, for *N* = 3 harvests, each in triplicate wells. 2-way ANOVA, with Bonferroni’s post hoc test, no significant differences. **g**, **h** TREM2-activating antibody stimulation (used at concentration of 2.4 μg/1 × 10^6^ cells and 3.84 μg/mL, for 10 min) of both WT and R47H TREM2 pMac caused SYK phosphorylation, measured by Western blotting. No response seen in TREM2 antibody-stimulated TREM2 KO cells, or cells treated with a goat IgG isotype control. **h** Means ± SEM, for *N* = 3 harvests measured on separate Western blots. 2-way ANOVA, with Sidak’s post hoc test, pairwise comparisons to WT for each treatment: *p* < 0.001 for KO stimulated with TREM2 Ab versus WT (****). **i** Calcium response in response to TREM2 antibody is similar in WT and R47H TREM2 pMac, measured by peak Fluo4-AM fluorescence, normalised to minimum fluorescence and cell number. Means ± SEM, for *N* = 3 harvests. 2-way ANOVA, with Dunnett’s multiple comparison test, pairwise comparisons to WT for each treatment: *p* = 0.0049 for KO stimulated with TREM2 Ab versus WT (**)

In this study, we aimed to characterise the effect of the R47H mutation on TREM2 protein expression, and on cell phenotypes, as illustrated in the diagram in Fig. 1a. TREM2 total protein levels in the R47H TREM2 line were unchanged relative to the WT line (Additional file 1: Fig. S3). TREM2 functions at the cell surface; therefore, proteins were purified by cell surface biotinylation and TREM2 detected by Western blotting, with comparison to the input cell homogenate (Fig. 1b). Surprisingly, the R47H TREM2 mutation showed significantly reduced cell surface levels of TREM2 by an average of 52% compared to the WT line in this assay (Fig. 1c). This difference in surface TREM2 was confirmed by immunocytochemistry of intact pMac (Additional file 1: Fig. S3), using an antibody that we confirmed is specific to TREM2 (unlike others, see Additional file 1: Fig. S4). Reduced surface TREM2 levels could be a consequence of either increased shedding from the membrane by regulated intra-membrane proteolysis [19, 44], TREM2 retention in the secretory pathway, or more TREM2 targeted to the lysosome for degradation. To distinguish these alternatives, we measured levels of TREM2 shedding by detecting the soluble TREM2 ectodomain (sTREM2) in conditioned media by ELISA and found that the R47H TREM2 pMac produced significantly higher levels of sTREM2 than WT in 4 h (Fig. 1d). This inverse relationship with cell surface TREM2 levels (Fig. 1c) supports the explanation that R47H increases TREM2 shedding. We also investigated TREM2 intracellular localization: immunocytochemistry of permeabilized pMac was used to measure co-localization of TREM2 with markers for the endoplasmic reticulum (ER), trans-Golgi network (TGN), and lysosomes. High levels of TREM2 in the TGN, and weak localization to the ER and lysosomes was observed; however, TREM2 localization between the WT and R47H pMac was not significantly different, although there was a trend towards reduced R47H TREM2 in the TGN (Fig. 1e, f). Taken together, the data suggests that R47H does not significantly alter TREM2 protein trafficking or maturation, but elevates shedding in pMac.

Stimulation of TREM2-DAP12 activates the kinase SYK [5], which can be identified by Western blotting with an antibody specific for double phosphorylation at Y525 and Y526 [38]. Additionally, SYK activation leads to release of intracellular calcium stores via phospholipase C, which can be measured by a live-cell fluorescent assay for calcium flux. To investigate functionality of the TREM2-DAP12 complex, a specific TREM2-activating antibody (AF1828, R&D Systems) was used to stimulate downstream signalling in pMac. The TREM2 antibody induced both SYK phosphorylation and calcium flux, which was absent in TREM2 KO pMac but unaffected by R47H TREM2, demonstrating that the R47H mutation does not alter TREM2 activity when a strong stimulus is applied (Fig. 1g–i, Additional file 1: Fig. S3).

### Several functional and morphological defects identified in TREM2 KO pMac are not exhibited by R47H TREM2 cells

#### Phagocytosis of dead SH-SY5Ys and synaptosomes is impaired in TREM2 KO but not R47H pMac

Loss of functional TREM2 has been previously reported to reduce microglial clearance of apoptotic neurons [6]. Phagocytosis of apoptotic neurons was modelled using two different phagocytic cargoes (for time-lapse videos, see Additional files 2 and 3): undifferentiated SH-SY5Ys that were freshly fixed with paraformaldehyde, and rat cortical synaptosomes, which are a synapse-enriched fraction of rat cortex (Additional file 1: Fig. S6). Fixing cells with paraformaldehyde has been previously reported to induce surface phosphatidylserine exposure, a signature of early apoptosis [40]. Both fixed (dead) SH-SY5Ys and synaptosomes were confirmed to expose surface phosphatidylserine, using annexin V staining (Additional file 1: Figs. S5, S6). An acid-sensitive dye, pHrodo iFL Red, was used to label the phagocytic cargoes to improve specific detection by high-content microscopy. Robust inhibition of the phagocytosis signal was achieved with actin inhibitors (cytochalasin D and jasplakinolide) and lysosome ATPase and autophagy inhibitor (bafilomycin A1), and significant enhancement by opsonising the cargo with human serum (Additional file 1: Figs. S5, Fig. S6). This demonstrates that the assay is sufficiently sensitive to discriminate alterations in phagocytic capability. Addition of recombinant annexin V to mask the exposed phosphatidylserine on the dead SH-SY5Ys led to a significant 30% reduction in phagocytosis, indicating that a large proportion of phagocytosis may be phosphatidylserine-independent (Additional file 1: Fig. S5).

**Fig. 2:**
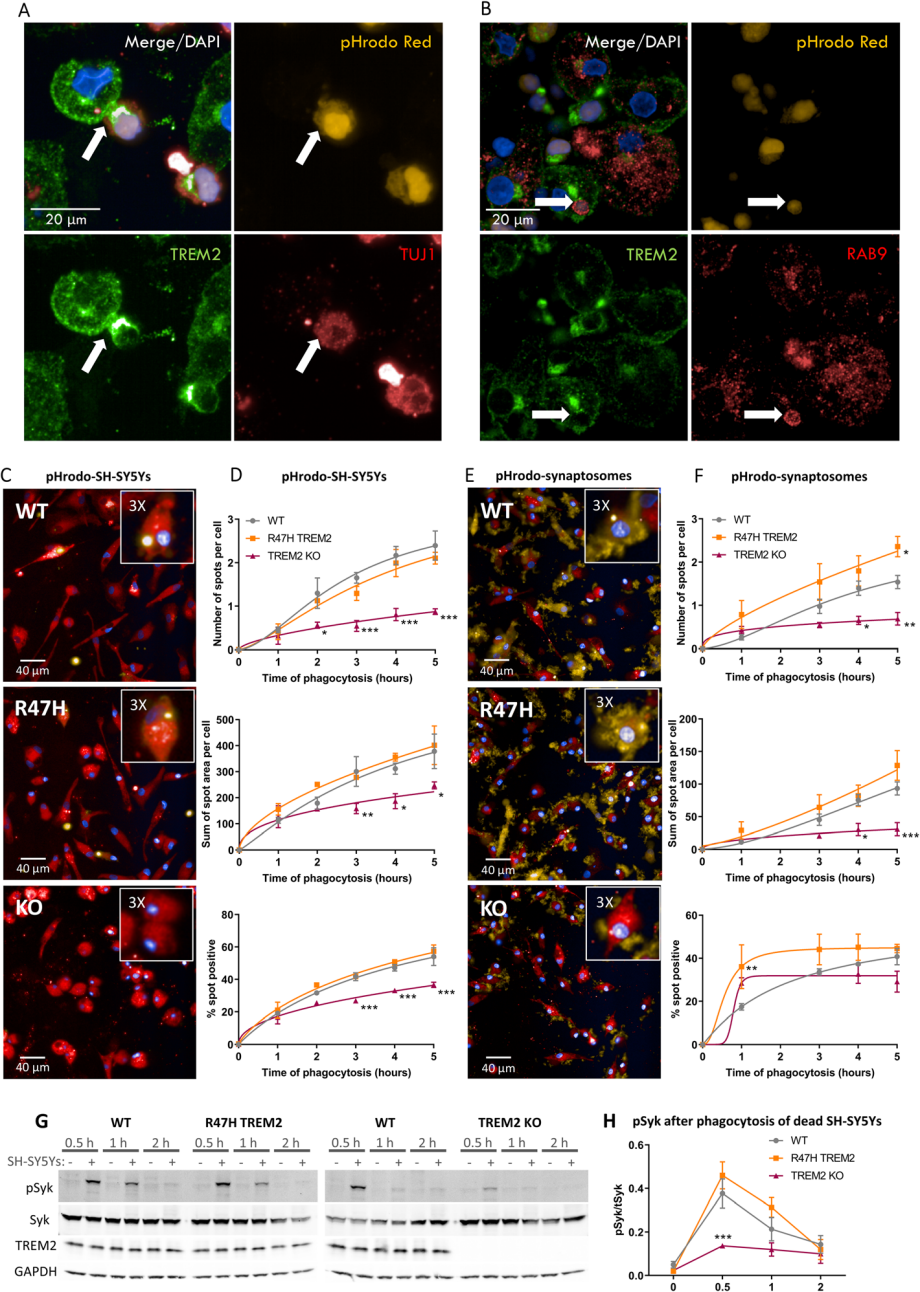
Phagocytosis of dead SH-SY5Ys and synaptosomes is reduced in TREM2 KO, but not R47H TREM2, relative to WT pMac. **a** After 3 h of phagocytosis of pHrodo-labelled dead SH-SY5Ys, immunofluorescence staining shows that TREM2 is highly recruited to the phagocytic cup (marked by white arrow) during engulfment of cells expressing the neuronal marker TUJ1, whereas in **b** TREM2 is lost before maturation to RAB9+ endosomes (marked by white arrow). **c**–**f** Phagocytosis is impaired in TREM2 KO pMac only. Representative images of phagocytosis of SH-SY5Ys (**c**) or synaptosomes (**e**) shown in yellow, by pMac (red cytoplasm and blue nucleus), taken at 3 h with INCell 6000. Inset is a section of the image magnified 3-fold. **d**, **f** Means were quantified for the parameters: number of spots per cell, sum of spot areas (μm^2^) per cell, percentage of cells containing phagocytosed particles per field. Data was normalised to mean for each genotype per experiment. Means ± SEM, for *N* = 3 harvests. Repeated-measures 2-way ANOVA, Dunnett’s post hoc test, pairwise comparisons to the WT for each time: *p < 0.05, **p < 0.01, ***p < 0.001. **g**, **h** Phagocytosis of dead SH-SY5Ys results in SYK phosphorylation, which is unaffected in R47H TREM2 cells but attenuated in TREM2 KO line, measured by Western blotting at 0.5, 1, and 2 h after phagocytosis initiation. **h** Means ± SEM, for *N* = 3 harvests. Repeated-measures 2-way ANOVA, with Dunnett’s post hoc test, pairwise comparisons to the WT for each time: *p* = 0.0008 at 0.5 h stimulation for KO versus WT (***)

The involvement of TREM2 in phagocytosis was investigated by performing ICC for TREM2 on control pMac phagocytosing pHrodo-labelled dead SH-SY5Ys. Strikingly, TREM2 was observed to be strongly concentrated at pMac phagocytic cups (Fig. 2a). Internalised SH-SY5Ys in RAB9+ endosomes had weak TREM2 signal, as would be expected for a phagocytic receptor that is quickly recycled back to the plasma membrane (Fig. 2b). TREM2 KO pMac had a 63% and 55% reduction in phagocytosis of dead SH-SY5Ys and synaptosomes respectively (Fig. 2c–f), in relation to the quantity of cargo consumed over 5 h (measured as average spots per well), indicating that these processes require TREM2. Additionally, a lower proportion of the TREM2 KO pMac phagocytosed dead SH-SY5Ys. With respect to synaptosome phagocytosis, the proportion of macrophages taking up cargo was saturated very quickly and therefore was not significantly reduced in the TREM2 KO line, likely due to the small size of the cargo (approximately 1.5 μm, measured by electron microscopy in Fig. S6, Additional file 1). In contrast, the R47H mutation of TREM2 did not significantly impair phagocytosis of dead SH-SY5Ys or synaptosomes, in fact there appeared to be an enhancement of synaptosome phagocytosis (Fig. 2f). Addition of dead SH-SY5Ys to the pMac in a time-course, with an unstimulated control for each time-point, produced a strong SYK phosphorylation signal in the WT line peaking around 30 min (Fig. 2g). SYK phosphorylation was not significantly different in the R47H line; however, the TREM2 KO had a significant 64% decrease in peak SYK phosphorylation relative to WT (Fig. 2h). The SYK phosphorylation response of the pMac to dead SH-SY5Ys is consistent with the levels of phagocytosis measured. Together, the data shows that recognition and engulfment of neuronal material by pMac is partially TREM2-dependent and that the R47H TREM2 mutant has no significant defect in this regard.

#### Altered morphology of TREM2 KO pMac is not mirrored by R47H pMac

**Fig. 3:**
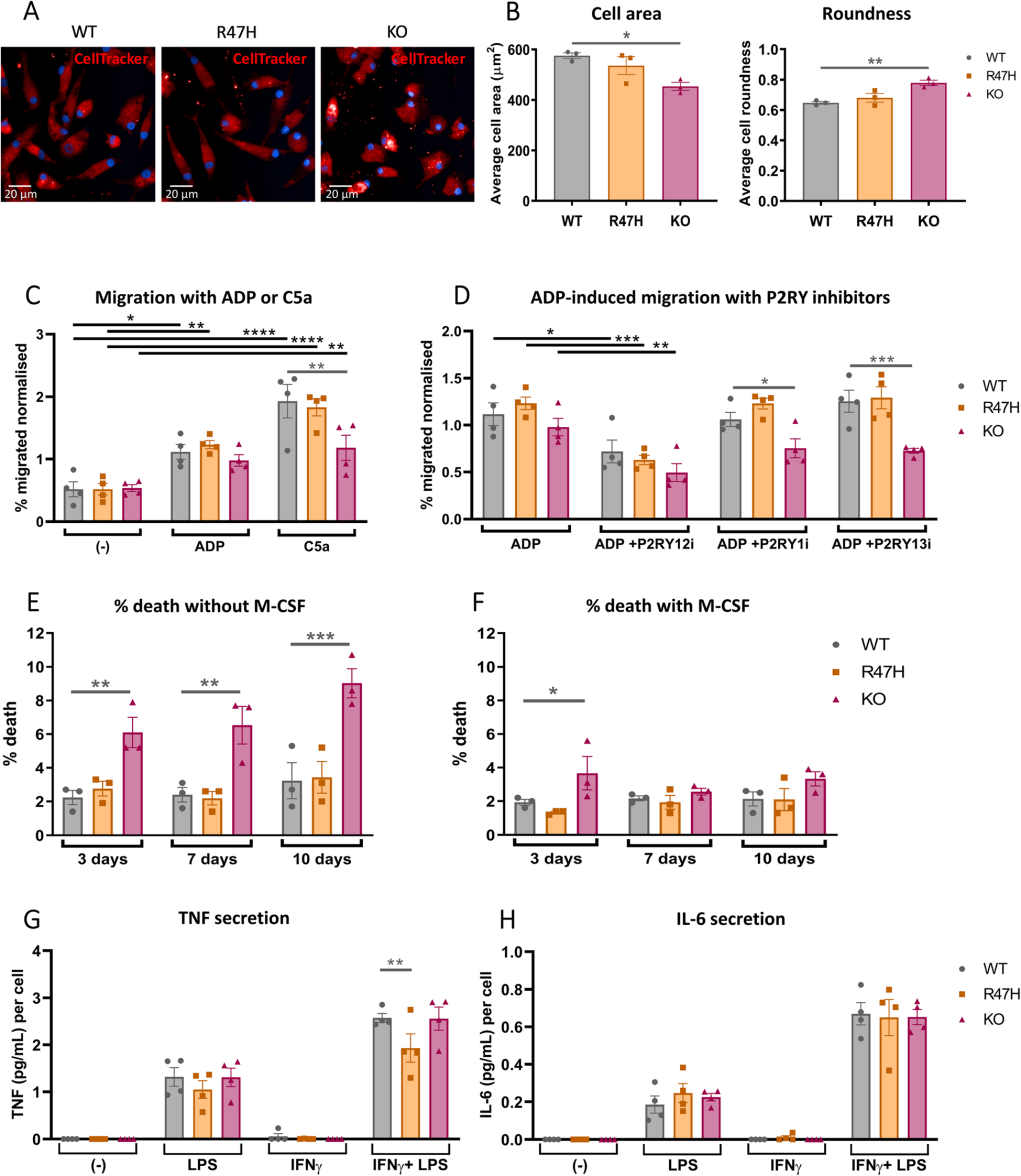
Divergent phenotypes in TREM2 KO and R47H TREM2 pMac, regarding cell morphology, migration, survival, and inflammatory responses. **a**, **b** TREM2 KO pMac are smaller and rounder than WT. Cell morphology measured by microscopy of pMac stained with CellTracker Deep Red, cells were fixed and imaged on INCell 6000 microscope. Representative images shown (**a**), and mean cell area (μm^2^) and roundness were automatically quantified from 9 fields per well in triplicate wells using Columbus software (**b**). Means ± SEM, for *N* = 3 harvests. 1-way ANOVA with Dunnett’s post hoc test: *p* = 0.019 for cell area in KO versus WT (*), *p* = 0.007 for roundness in KO versus WT (**). **c**, **d** TREM2 KO pMac migrate slower towards C5a, but not ADP, compared with WT. Cell migration measured by transwell assay. Migration of pMac towards 30 μM ADP or 3 nM C5a is compared to unstimulated migration over 6 h (**c**), and inhibitors used to unmask the contribution of purinergic receptors P2RY1 (3 μM MRS2179), P2RY12 (30 μM PSB0739), and P2RY13 (10 μM MRS2211) to ADP-induced migration (**d**). Data is expressed as the percentage of migrated cells and was normalised to average migration for the harvest. Means ± SEM, for *N* = 4 harvests. 2-way ANOVA with Dunnett’s post hoc test. Black annotations compare stimulation to unstimulated control, or ADP + purinergic inhibitors to the ADP-only control. Grey annotations compare R47H or KO versus WT for each stimulation. * *p* < 0.05, ** *p* < 0.01, *** *p* < 0.001, **** *p* < 0.0001, all unannotated comparisons are not significant. **e**, **f** TREM2 KO pMac exhibit increased cell death in the absence of M-CSF, whereas WT and R47H pMac remain viable. Survival after M-CSF withdrawal measured at 3, 7, and 10 days, with a comparison between M-CSF-deficient condition (**e**) to full-media controls (**f**) in the same plate. Means ± SEM, for *N* = 3 harvests. Repeated-measures 2-way ANOVA, with Dunnett’s post hoc test, pairwise comparisons to WT for each time: M-CSF-deficient KO versus WT at 3 days *p* = 0.0051 (**), at 7 days *p* = 0.003 (**), and at 10 days *p* = 0.0001 (***). Full media KO versus WT at 3 days *p* = 0.035 (*). **g**, **h** Comparable secretion of TNF and IL-6 by pMac in response to *E. coli* LPS (100 ng/mL, 4 h) ± priming by interferon-γ (100 ng/mL, 24 h prior to LPS). Concentration of TNF (**g**) and IL-6 (**h**) was measured by separate ELISAs of the same supernatants, normalised to cell number, and normalised to the average pg/mL/cell for the harvest. Means ± SEM, for *N* = 4 harvests. 2-way ANOVA, with Dunnett’s post hoc test, pairwise comparisons to WT for each stimulation: *p* = 0.0097 for TNF secreted from R47H versus WT with LPS ± IFNγ stimulation (**). As expected, IFNγ-priming enhances TNF and IL-6 secretion upon LPS stimulation, significance is not depicted for clarity, but *p* < 0.001 for IFNγ+LPS versus LPS, for TNF and IL-6 of all genotypes

Altered morphology of the TREM2 KO pMac relative to the WT line was consistently observed during measurements of phagocytosis. From the phagocytosis negative control images of pMac, with cytosolic and nuclear fluorescent stains, two-dimensional cell morphological parameters were calculated using Columbus software. TREM2 KO pMac were significantly smaller and rounder than WT pMac, whereas R47H TREM2 pMac were not significantly different to the WT (Fig. 3a, b).

#### Directed migration is impaired in TREM2 KO but not R47H pMac

Migration defects have been previously identified in TREM2 KO mice, with Ccl2, C5a, or apoptotic neurons as chemotactic stimuli [45]. Migration of pMac was measured by plating into transwell inserts with 5-μm pores, and after 6 h, the percentage of cells that had moved to the underside of the inserts was measured. Migration increased in a dose-dependent manner with ADP or C5a spiked into media below the transwells (Additional file 1: Fig. S7; Fig. 3c). ADP, released by apoptotic cells, causes mainly chemokinesis, whereas C5a is a potent chemotaxis inducer [46, 47]. In the TREM2 KO pMac, ADP caused a similar increase migration to the WT (Fig. 3c). However, C5a-stimulated migration was significantly impaired, with an average reduction of 40% (Fig. 3c). The R47H TREM2 cells had no impairment of ADP-induced or C5a-induced migration (Fig. 3c). Interestingly, further investigation of ADP-stimulated migration with P2Y receptor inhibitors revealed that TREM2 KO pMac rely upon multiple P2Y receptor sub-types for ADP detection, whereas the WT and R47H lines mainly require P2RY12 activity (Fig. 3d). Selective inhibition of P2RY12 receptors by PSB0739 reduced ADP-stimulated migration in all genotypes, but a P2RY1 inhibitor (MRS2179) and a P2RY13 inhibitor (MRS2211) reduced migration of only TREM2 KO cells. RNA-seq can help to explain this phenomenon: transcription of P2RY1 and P2RY13 was significantly increased in unstimulated TREM2 KO pMac relative to the WT (Fig. 5b), whereas P2RY12 was not differentially expressed. Despite upregulated purinergic receptor expression, the TREM2 KO cells had similar ADP-stimulated migration to the WT, suggesting that increased ADP detection is counterbalanced by a generalised motility defect. Taken together, the data suggests defective migration in TREM2 KO pMac, whereas the R47H TREM2 pMac had similar migration to the WT under all conditions tested.

#### Survival after M-CSF withdrawal is impaired in TREM2 KO but not R47H pMac

TREM2 activates AKT and β-catenin-mediated pro-survival signalling and is proposed to have tonic activity in the absence of any specific damage-associated or pathogen-associated signals [5, 48]. The growth factor M-CSF, used for macrophage differentiation, stimulates the same pro-survival signalling pathways and therefore could compensate for TREM2 deficiency unless depleted. After 7 days of differentiation with M-CSF, M-CSF was withdrawn from pMac by a full media change, and cell death analysed after a further 3–10 days in comparison to complete media. Interestingly, WT and R47H TREM2 pMac did not lose viability in response to M-CSF withdrawal, even after 10 days (Fig. 3e). There was a small but significant enhancement of cell death following M-CSF withdrawal in TREM2 KO pMac that increased over time. In full media, there was little change in viability over time (Fig. 3f); therefore, cell death was largely driven by M-CSF withdrawal.

#### LPS-induced inflammation is not strongly perturbed in TREM2 KO or R47H pMac

TREM2 deficiency has been reported to potentiate bacterial lipopolysaccharide (LPS)-induced inflammation in mouse bone marrow-derived macrophages, but not human microglia-like cells [49–51]. Challenge with *E. coli* LPS dose-dependently upregulated secretion of the proinflammatory cytokines TNF and IL-6, in pMac from all three genotypes (Additional file 1: Fig. S7). Only at the highest dose of LPS, the TREM2 KO line produced significantly less TNF than the WT line, whereas IL-6 secretion was unaltered. When the pMac were primed with IFNγ for 24 h before stimulation with LPS (Fig. 3g, h), clear potentiation of TNF and IL-6 responses was observed as expected [50]. Here, cytokine secretion of the TREM2 KO cells was unchanged versus the WT line, whereas in the R47H TREM2 line there was a significant (25%) reduction of TNF secretion. Together, these results suggest that TREM2 expression is not acting as a brake on inflammation in pMac.

### RNA sequencing reveals shared transcriptional signature of the TREM2 KO and R47H TREM2

**Fig. 4:**
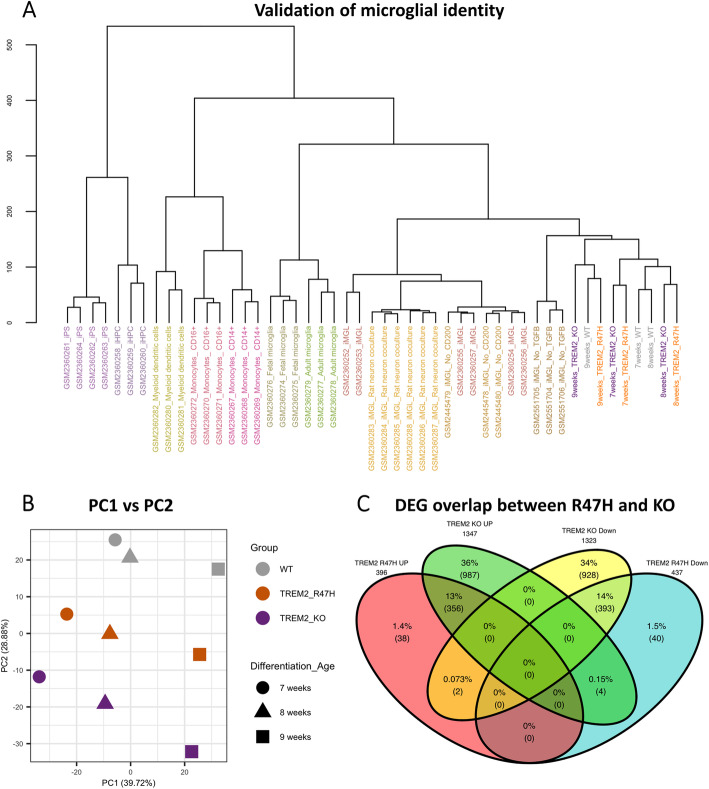
Transcriptomics reveal high overlap in dysregulated genes of R47H TREM2 and TREM2 KO pMac, relative to WT. **a** Validation of pMac “microglial identity” by dendrogram comparison to Abud et al. transcriptomes of iPS cells, iPSC-haematopoietic progenitor cells (iHPC), iPSC-microglia-like cells (iMGL), iMGL without TGFβ1 or without CD200 supplementation, iMGL co-cultured with rat cortical neurons, blood-derived monocytes, blood-derived dendritic cells, and primary foetal and adult human microglia [42]. **b** Plot of principal component analysis (PCA) with the first two principal components separating the RNA-seq samples by differentiation age and genotype. Ages represented by shapes, and genotypes represented by colour. **c** Venn diagram of differentially expressed genes (DEGs) identified relative to the WT line, showing the overlap between R47H TREM2 and TREM2 KO DEGs. DEGs are separately categorised as “upregulated” or “downregulated” relative to the WT

Although no gross phenotypic abnormalities were detected in the R47H TREM2 pMac, more subtle effects of the mutation upon cell processes would be signposted in the transcriptional signature. We performed RNA sequencing (RNA-seq) of unstimulated pMac from three consecutive weeks of harvests, comparing the TREM2 KO and R47H lines with WT. We firstly confirmed the microglial identity of the cells by comparing their transcriptome with data from Abud et al., who used cells of myeloid lineages including primary microglia and iPSC-microglia-like cells (Fig. 4a) [42]. Our pMac cluster closely with the Abud et al. iPSC-microglia-like cells, and their *TREM2* genotype has no effect on clustering in this context; instead, there is a little separation by differentiation age. Our pMac, which are not exposed to exogenous TGFβ, are most similar to the Abud et al. samples with TGFβ withdrawal. Next, we performed principal component analysis to identify sources of transcriptional variation. Interestingly, the differentiation age of the cells harvested at weekly intervals accounted for nearly 40% of the variation on PC1, whereas *TREM2* genotype accounted for 29% of the variation (PC2) (Fig. 4b). On a PCA plot of PC1 versus PC2, the samples are ordered along the PC2 axis as KO-R47H-WT, showing clearly that the R47H mutation has an intermediate transcriptional impact in comparison to TREM2 KO. It is also clear that strong age-related changes in gene expression could be confounding; therefore, PC1 was added as a covariate to the design formula for identifying differentially expressed genes.

**Fig. 5:**
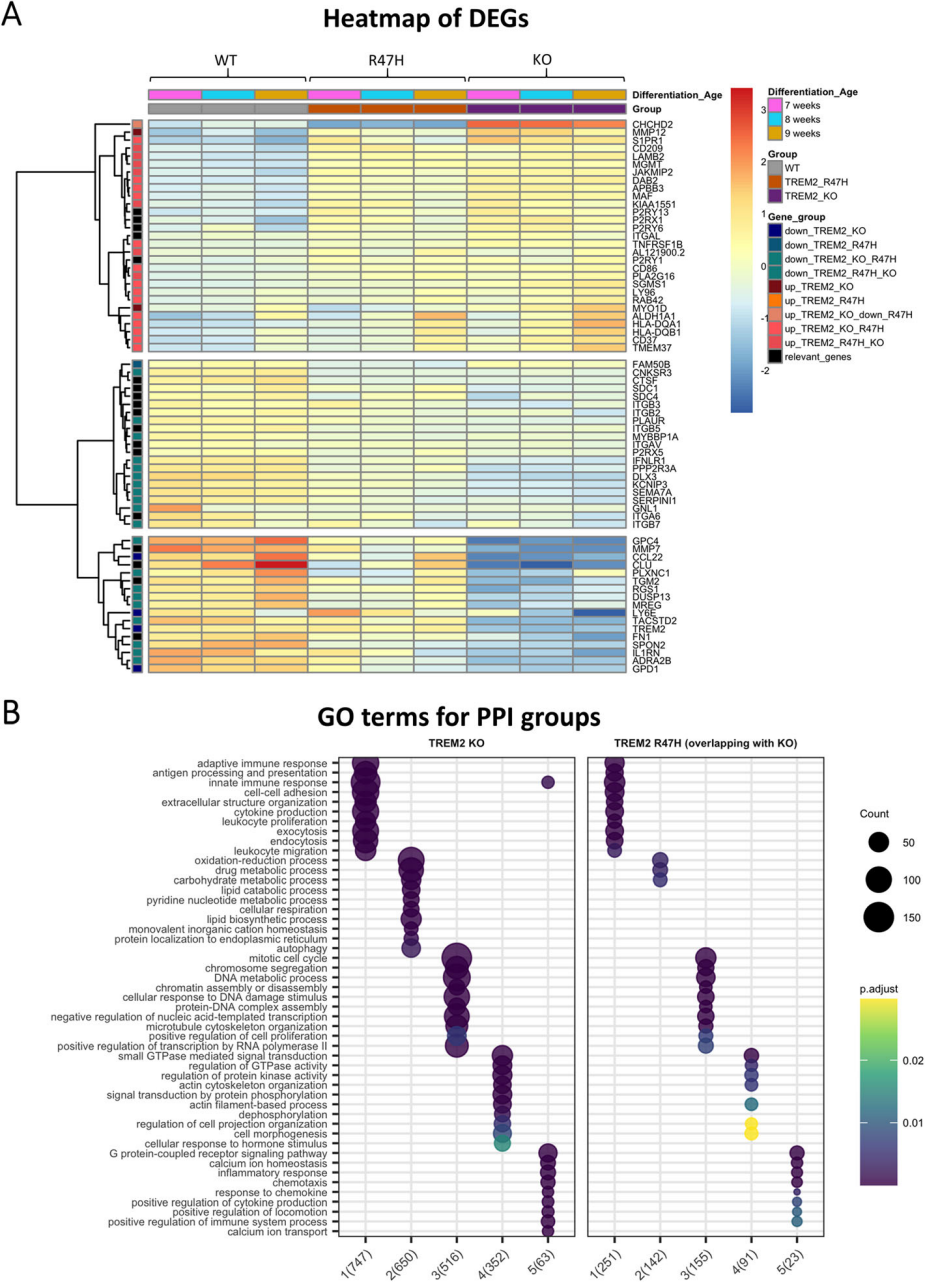
Top DEGs of R47H TREM2 and TREM2 KO pMac overlap; furthermore, the R47H DEGs that are shared with TREM2 KO represent numerous biological processes, distributed between five protein-protein interaction (PPI) modules. **a** Heatmap of top upregulated and top downregulated DEGs for R47H TREM2 and TREM2 KO, with the relative expression for each gene represented by colours on the corresponding row. A selection of “relevant genes” was also included. Unbiased clustering (dendrogram on the left) shows that genes separate into two major clusters based on whether they are upregulated or downregulated in TREM2 KO or R47H TREM2, and there is no genotype-specific segregation. **b** Five modules of functionally related DEGs identified by PPI network analysis of TREM2 KO DEGs, with enriched gene ontology (GO) terms shown. R47H DEGs were only included where they were also differentially expressed in TREM2 KO and were overlaid onto clusters identified using TREM2 KO data. Clusters identified numerically on the *x*-axis, with TREM2 KO on the left and R47H TREM2 on the right, showing a similar pattern of dysregulated cell functions. Number of DEGs represented by circle size, and *p* value represented by colour

Significantly differentially expressed genes (DEG) were then identified relative to the WT pMac (adjusted *p* value < 0.05), with 2670 in the TREM2 KO versus WT and 833 in the R47H TREM2 versus WT. Top DEGs for TREM2 KO and R47H are illustrated as volcano plots in the supplementary data (Additional file 1: Fig. S8), and as a heatmap in Fig. 5a, with the inclusion of some extra DEGs selected for their relevance to cell motility and adhesion. A Venn diagram shows that 90% of DEGs identified in the R47H TREM2 line are also differentially expressed in the TREM2 KO, with the same direction of regulation (Fig. 4c). Only 6 of the 833 R47H TREM2 DEGs are regulated in a different direction to the TREM2 KO: *CHCHD2*, *THRB*, *ZNF248*, and *TRIM4* are down in the R47H and up in the KO; *ST3GAL1* and *S100A1* are up in the R47H and down in the KO. Gene ontology (GO) analysis for KO and R47H DEGs revealed enrichment for immunity, inflammation, proliferation, migration, and adhesion (Additional file 1: Fig. S8). To further explore the dysregulated functional pathways, we performed an unbiased Louvain clustering of the TREM2 KO DEGs in protein-protein interaction (PPI) networks, to identify 5 different modules of functionally related genes, and identified the top 10 most enriched GO terms for each group (Fig. 5b). Group 1 is the largest group and associated with immune responses, cell-matrix adhesion, migration, proliferation, endocytosis, and exocytosis. Group 2 is associated with metabolic processes including lipid and carbohydrate metabolism and also includes autophagy. Group 3 is associated with cell division, whereas group 4 is associated with actin organisation and GTPase and kinase regulation. Group 5 is small and includes terms for chemotaxis and calcium ion homeostasis. We then overlaid the R47H TREM2 DEGs that overlap with TREM2 KO onto the same PPI groups, in order to determine whether the R47H dysregulated genes are spread over many GO terms or confined to a single biological process (Fig. 5b). The results confirmed that the R47H DEGs hit almost every biological process that the TREM2 KO DEGs target, and therefore that the R47H mutation causes a partial loss of function of TREM2.

### Cell adhesion to extracellular matrix is dysregulated by R47H TREM2 and TREM2 KO, and role of TGF-β1 was investigated

**Fig. 6:**
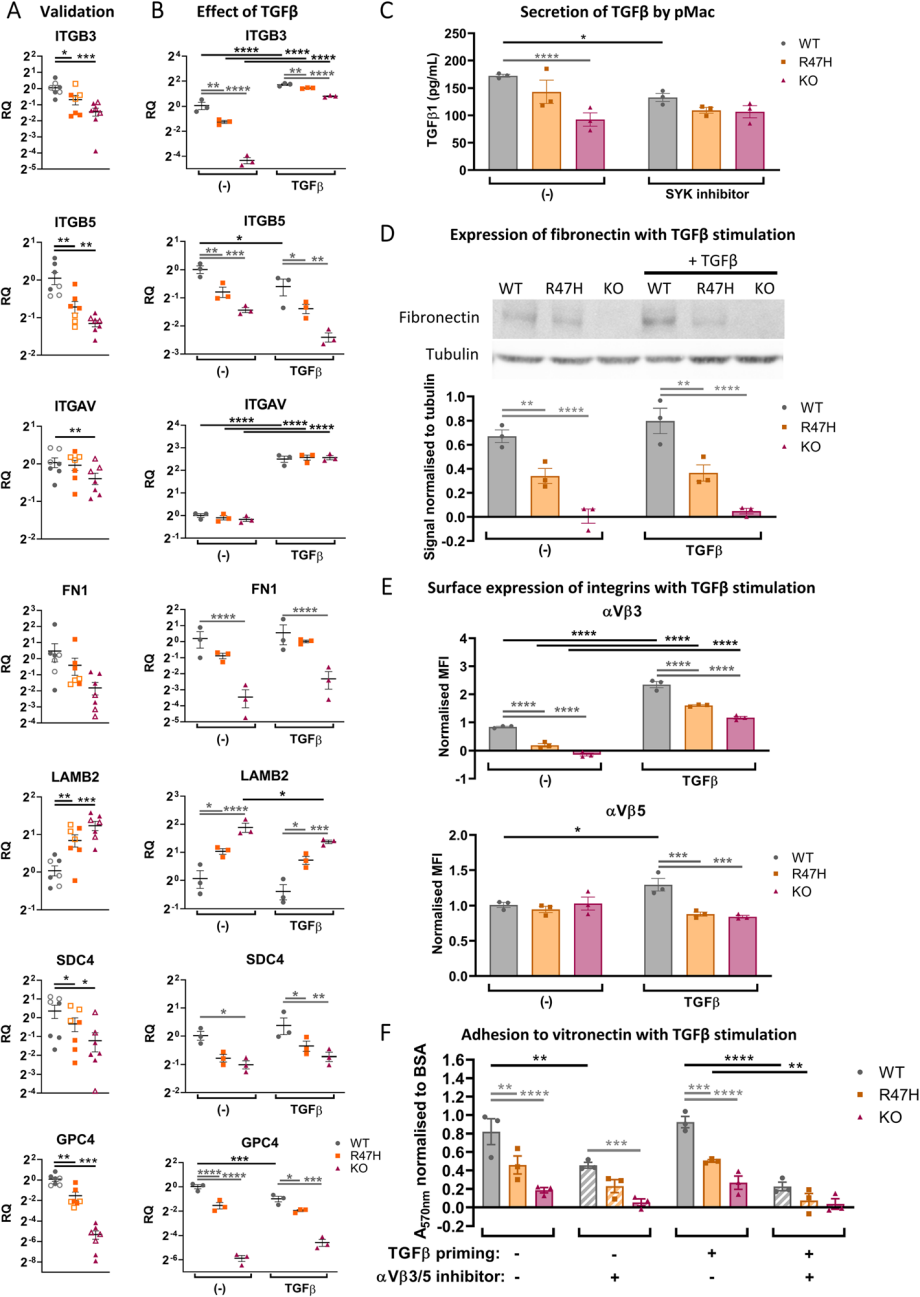
Extracellular matrix-adhesion modifiers and adhesion to vitronectin are dysregulated in R47H TREM2 and TREM2 KO pMac. TGFβ treatment does not rescue adhesion deficits. **a** Validation of selected RNA-seq hits by qRT-PCR of unstimulated pMac. Means ± SEM, for *N* = 7 harvests, including the 3 samples originally used for RNA-seq (open symbols) plus 4 samples harvested independently from a separate differentiation (filled symbols). Repeated-measures 1-way ANOVA, with Dunnett’s post hoc test, pairwise comparisons to the WT. **b** Effect of TGFβ-stimulation (50 ng/mL, 24 h) on mRNA levels of selected RNA-seq hits, measured by qRT-PCR. Means ± SEM, for *N* = 3 samples harvested independently to Fig. 5a. Two-way ANOVA, with Sidak’s post hoc test. **c** TREM2 KO pMac secrete reduced levels of TGFβ1 compared with WT, and TGFβ1 secretion is partly SYK-dependent. Total (inactive and active) TGFβ1 measured from supernatants by ELISA, cells treated ± OXSI-2 (2 μM, 24 h) to inhibit SYK. Means ± SEM, for *N* = 3 harvests. Two-way ANOVA, with Sidak’s post hoc test. **d** Fibronectin protein expression is reduced in both R47H TREM2 and TREM2 KO versus WT. Fibronectin measured by Western blotting of pMac ± TGFβ1 stimulation (50 ng/mL, 24 h). Means ± SEM, quantified for *N* = 3 harvests on separate blots. Two-way ANOVA, with Sidak’s post hoc test. TGFβ1 vs unstimulated was not significant. **e** αVβ3 complex formation is reduced in both R47H TREM2 and TREM2 KO versus WT. Intact surface integrins αVβ3 and αVβ5 measured by flow cytometry in pMac ± TGFβ1 stimulation (50 ng/mL, 24 h). Data is expressed as the difference in median fluorescence intensity of the specific antibody versus isotype control, normalised (by subtraction) to the average for the harvest. Means ± SEM, for *N* = 3 harvests. Two-way ANOVA, with Sidak’s post hoc test. **f** Adhesion to vitronectin is reduced for both R47H TREM2 and TREM2 KO versus WT, and treatment with TGFβ (50 ng/mL, 24 h prior to assay) increases αVβ3/5-dependent adhesion. Adhesion measured after 3 h by crystal violet colorimetric assay, and normalised to BSA-blocked wells (by division, and the result subtracted from 1). αVβ3/5 inhibitor (10 μM cilengitide) was added at the start of the assay to determine αVβ3/5-specific adhesion to vitronectin (striped bars). Means ± SEM, for *N* = 3 harvests. Two-way ANOVA, with Dunnett’s post hoc test. Black annotations compare stimulations to unstimulated control. Grey annotations compare R47H or KO versus WT for each condition. WT = grey circles, R47H = orange squares, TREM2 KO = burgundy triangles. *p < 0.05, **p < 0.01, ***p < 0.001, ****p < 0.0001, all unannotated comparisons are not significant

Transcript levels of selected adhesion-related DEGs were validated by qRT-PCR, using four harvests of pMac from another differentiation, compared directly with samples of extracted RNA used for the RNA-seq (three harvests). There was strong overlap between the original RNA-seq samples (open symbols) and samples obtained subsequently (filled symbols), for all of the genes: *ITGB3*, *ITGB5*, *ITGAV*, *FN1*, *LAMB2*, *SDC4*, and *GPC4* (Fig. 6a). *ITGB3*, *ITGB5*, *SDC4*, and *GPC4* were significantly downregulated in both the R47H and KO pMac. *ITGAV* was significantly downregulated in the KO but not R47H pMac. *LAMB2* was significantly upregulated in both the R47H and KO pMac. *FN1* was not significantly downregulated in this experiment, but in an independent follow-up assay, there was a significant reduction in TREM2 KO pMac (Fig. 6b).

We then used the Ingenuity Pathways Analysis (IPA) platform to search for common predicted upstream regulators, within each of the previously identified functionally related modules. Due to the large size of group 1 module and its relevance to migration and adhesion phenotypes, we were particularly interested in top hits for group 1, shown in Tables 2 (inhibitory) and 3 (activatory). The group 1 top hits predicted to inhibit the TREM2 KO transcriptional signature included TGFβ1 (Table 2). TGFβ1 is a growth factor commonly used for maintenance of primary microglia and of iPS-microglia-like cell cultures [42, 52], but not included in our culture. We stimulated pMac with recombinant human TGFβ1 for 24 h, to assess whether TGFβ1 could “rescue” expression of the aforementioned selected genes (Fig. 6b). TGFβ1 treatment strongly induced expression of *ITGB3* and *ITGAV*, effectively rescuing the effect of the TREM2 mutations. TGFβ1 also diminished *LAMB2* expression in TREM2 KO pMac, although not down to the same level as WT. However, the other genes were either unaffected by TGFβ1 (*CHCHD2*, *FN1*, *SDC4*), or downregulated in the WT line, mimicking the TREM2 mutations (*ITGB5*, *GPC4*).

**Table 2:** Top 5 upstream regulators predicted to oppose TREM2 KO (group 1 genes)

Name of regulator	Synonyms	Function	Activation *Z*-score	*p* value of overlap	Number of group 1 genes
Dexamethasone	–	Corticosteroid drug	− 2.66	1 × 10^−38^	195
Transforming growth factor-β	TGFβ1	Growth factor	− 2.55	7 × 10^− 25^	149
Filgrastim	filgrastim-aafi, filgrastim-sndz	Recombinant human G-CSF drug	− 2.40	5 × 10^− 22^	62
Immunoglobulin	Ig, antibody	Adaptive immunity	− 2.56	1 × 10^−16^	69
Mitogen activated protein kinase 1	MAPK1, ERK2, p42MAPK	Kinase downstream of growth factor receptors	− 2.89	2 × 10^−16^	50

Upstream regulators predicted by IPA to be inhibitory, ordered by lowest *p* value

**Table 3:** Top 5 upstream regulators predicted to mimic TREM2 KO (group 1 genes)

Name	Synonyms	Function	Activation *Z*-score	*p* value of overlap	Number of group 1 genes regulated
Interleukin-10	IL10(A), CSIF, GVHDS, TGIF	Cytokine	+ 2.13	8 × 10^− 22^	60
Interleukin-27	IL27(A), IL30, P38	Cytokine	+ 2.57	2 × 10^− 21^	36
Fas receptor	FAS, FasR, FASTM, ALPS1A, APO-1, APT1, CD95, TNFRSF6	Receptor for Fas ligand, initiates apoptosis	+ 2.44	3 × 10^− 21^	56
Interferon-α2	IFNA2(A/B/C)	Cytokine, type I interferon	+ 3.46	2 × 10^−19^	43
Signal transducer and activator of transcription 1	STAT1, ISGF-3, CANDF7, IMD31(A/B/C), STAT91	Transcription factor downstream of interferon receptors	+ 2.48	2 × 10^−15^	47

Upstream regulators predicted by IPA to be activatory, ordered by lowest *p* value

Since TGFβ1 was predicted to oppose some of the transcriptional changes wrought by TREM2 KO, we hypothesised that TREM2 KO pMac are expressing less TGFβ1 protein than WT and that loss of TGFβ1 may mediate cell dysfunction in TREM2 KO pMac. TGFβ1 transcripts are not significantly downregulated in the RNA-seq. However, TGFβ1 protein secretion from pMac, measured by ELISA, is significantly reduced in TREM2 KO pMac relative to the WT (Fig. 6c). The R47H pMac do not have significantly different TGFβ1 secretion to WT. Furthermore, incubation with a SYK inhibitor mimicked the effect of TREM2 KO (Fig. 6c), which suggests that reduced SYK activation in TREM2 KO pMac could reduce TGFβ1 expression.

Next, we looked to validate protein expression for *FN1, ITGAV*, *ITGB3*, and *ITGB5*, which encode fibronectin, αV-integrin, β3-integrin, and β5-integrin respectively. Fibronectin protein was measured from whole-cell lysates by Western blotting and is significantly reduced in both R47H TREM2 and TREM2 KO pMac (Fig. 6d), consistent with the mRNA levels (Fig. 6b). Similarly, treatment of cells with recombinant TGFβ1 did not rescue fibronectin protein expression, which is consistent with the mRNA levels (Fig. 6b). Functional surface integrin complexes of αVβ3 and αVβ5 were detected by flow cytometry. Integrin αVβ3 was downregulated in R47H TREM2 pMac, relative to WT and was further downregulated in TREM2 KO pMac (Fig. 6e), consistent with the mRNA levels (Fig. 6a, b). Surprisingly, levels of αVβ5 in R47H TREM2 and TREM2 KO pMac were comparable to WT (Fig. 6e), despite having lower transcription of *ITGAV* and *ITGB5* (Fig. 6a, b). We also tested the effect of TGFβ1 stimulation upon expression of αVβ3 and αVβ5 (Fig. 6e; Additional file 1: Fig. S9). TGFβ1 treatment for 24 h (50 ng/mL) caused dramatic upregulation of αVβ3 integrins at the cell surface, rescuing expression in the R47H TREM2 and TREM2 KO pMac. Conversely, surface αVβ5 was upregulated by TGFβ1 only in the WT pMac and not in the R47H TREM2 and TREM2 KO pMac. Collectively the protein expression data for fibronectin, αVβ3, and αVβ5 largely agree with the transcriptional dysregulation identified by RNA-seq and qRT-PCR, but demonstrate that only αVβ3 is strongly upregulated by TGFβ1 stimulation.

Finally, we sought to ascertain whether TGFβ1 could rescue phenotypic defects of the TREM2 KO pMac. We hypothesised that αVβ3 expression would impact the adhesion of pMac to vitronectin. Mouse microglia adhere to vitronectin using the integrins αVβ3 and αVβ5, probably with contributions from other αV integrins [53], and additionally the urokinase receptor (uPAR) may be involved [54]. Adhesion of pMac to vitronectin was measured by a 96-well plate colorimetric assay, and the contribution of αVβ3/5 integrins inhibited by treatment with cilengitide. Total vitronectin adhesion of unstimulated cells (Fig. 6f, first set of bars, between genotypes) was significantly reduced by 44% in R47H TREM2 and 77% in TREM2 KO pMac compared to WT. Compared with total adhesion, the αVβ3/5 inhibitor reduced adhesion to vitronectin by 56% in WT (significant), indicating that αVβ3/5 has a large contribution to vitronectin adhesion (Fig. 6f, first and second sets of bars). Interestingly, in the presence of αVβ3/5 inhibitor, there was significantly less adhesion of TREM2 KO versus WT pMac (Fig. 6f, second set of bars between genotypes). This suggests that other types of vitronectin-adhesion molecule are also reduced by TREM2 KO, e.g., uPAR. Our RNA-seq data supports a significant downregulation of uPAR in both R47H and TREM2 KO pMac, encoded by the *PLAUR* gene. TGFβ1 treatment of pMac for 24 h prior to the assay did not significantly increase total vitronectin adhesion (Fig. 6f, first and third set of bars); however, the αVβ3/5-specific adhesion appears to be increased (Fig. 6f, third set of bars between genotypes)). The αVβ3/5 inhibitor reduced adhesion of TGFβ1-treated cells by 76% in WT (significant), 85% in R47H (significant), and 86% in TREM2 KO (not significant) (Fig. 6f, third and fourth sets of bars). The αVβ3/5-specific adhesion of TGFβ-treated cells appears to be much higher than non-primed cells. Our interpretation of this data is that TGFβ1 treatment promotes αVβ3 expression and ligation with vitronectin, but is not sufficient to rescue adhesion of TREM2 mutant cells, perhaps due to defective expression of other adhesion molecules and cytoskeletal regulators that are not TGFβ-sensitive.

## Discussion

The primary aim of this study was to identify phenotypic defects caused by the R47H TREM2 mutation in a human microglia model in vitro. Although the TREM2 KO pMac exhibited significant defects of phagocytosis, migration, and survival, the R47H mutation had too modest an effect on TREM2 activity to disturb the same broad cell functions. Identifying dysregulated genes with transcriptomics allowed us to design targeted cell assays and led to the discovery that R47H TREM2 significantly impairs adhesion to vitronectin, a small-scale phenotypic defect that could nonetheless be important. The general lack of gross phenotypic defects is curious in the context of previous literature. Human AD patients carrying the TREM2 risk variants have more dystrophic microglia, weaker interaction of microglia with amyloid plaques, increased dystrophic neurites, and larger areas of insoluble phosphorylated tau than age- and disease stage-matched AD patients without an AD risk variant [55, 56]. The implications of this are that TREM2 AD risk variant microglia are dysfunctional, although the precise mechanistic details have not been defined. With regard to the lack of gross phenotypic defects, clearly there are three major components missing from our in vitro model compared with human AD brains—ageing, environment, and interactions with other cell types—and the pathogenic effects of the R47H TREM2 mutation may be more evident in combination with these missing components.

### Effect of R47H on TREM2

We firstly validated TREM2 expression and activity in the R47H TREM2 pMac and identified a reduction in cell surface expression of TREM2 and increased shedding compared with isogenic WT pMac. This finding is supported by a study of AD patients’ cerebrospinal fluid, which showed that p.R47H carriers had higher sTREM2 levels than non-carriers [57]. However, another study of human AD brain cortices, examining detergent-extracted C-terminal fragments of TREM2 as a proxy for TREM2 shedding, found no difference between p.R47H and non-carriers [58]. A potential explanation for this is that the R47H TREM2 mutation is always heterozygous when identified in human AD patients, and therefore, the overall impact on TREM2 surface expression is small. We used a homozygous R47H TREM2 line to offer the maximum opportunity to detect potential phenotypic defects, without compensation from a normal allele, although this strategy has the limitation of not replicating the mutation dosage associated with the monoallelic R47H mutation common in human AD; therefore, the impact of the mutation on phenotype could be overstated in biallelic cells. Three previous studies found no abnormality of R47H TREM2 cell surface expression in cell cultures; however, these either overexpressed human TREM2 in HEK293 cell lines [19, 59] or used gene-edited mice that express murine TREM2 [60], which undergoes abnormal splicing when R47H is inserted [25]. On the other hand, changes to TREM2 complex glycosylation, and reduced stability and retromer-mediated receptor recycling have been detected in transfected HeLa cells [61, 62]. Although most sTREM2 is produced by proteolytic cleavage of the full-length TREM2 protein, about 25% in human brains is estimated to originate from expression of a shorter splice isoform of TREM2 that lacks the transmembrane domain (ENST00000338469) [63, 64]. To ascertain whether the increased sTREM2 production of R47H TREM2 cells originates from a short isoform of TREM2, we performed differential transcript usage analysis on our RNA-seq dataset (Additional file 1: Fig. S10). There was no significant difference to the use of TREM2 alternative transcripts in the R47H line compared with WT, which supports previous literature [63]. In the literature, there is no direct connection between R47H and TREM2 shedding, but we speculate that reduced TREM2 ligand engagement could allow sheddases greater access to TREM2, whereas in the ligated state, TREM2 may be inaccessible to sheddases. One potential mechanism is that ligated TREM2-DAP12 complexes may segregate to “lipid rafts” in the plasma membrane [65, 66], whereas ADAM10 is thought to be excluded from lipid rafts [67]. Another possibility is that tight “immunological synapses” form between the iPSC-macrophage and TREM2-activating target membranes, e.g., dead cells, excluding proteins with larger extracellular domains such as ADAM10. Future work to distinguish the two mechanisms could include co-localization studies of ADAM10 with TREM2 and lipid raft markers. Nevertheless, despite reduced cell surface expression of TREM2 in R47H TREM2 pMac, robust downstream signalling was provoked by a TREM2-activating antibody and by dead neurons, demonstrating no significant reduction in downstream SYK and calcium signalling (Figs. 1h, i and 2h).

### Previous literature on effect of R47H TREM2 on microglia function

The TREM2-dependent phenotypes that we have published with TREM2 KO pMac are in strong agreement with previous literature. iPSC-derived microglia-like cells with either TREM2 KO or severe loss-of-function mutations (T66M, W50C) have reduced phagocytosis of apoptotic neurons and amyloid plaques, and poorer survival after M-CSF withdrawal, but no significant defect in TLR4-mediated cytokine responses [11, 27, 51, 68]. Claes et al. generated the first published R47H mutation in human embryonic stem cells and showed that it had no impact on phagocytosis of ex vivo amyloid plaques or *E. coli* by microglia-like cells, whereas TREM2 ^−/+^ and ^−/−^ cells had defective phagocytosis of these cargoes, agreeing with our findings that R47H does not phenocopy TREM2 deficiency in human microglia models [27]. We show that the R47H mutation attenuates IFNγ-primed LPS inflammation, an observation that is also in agreement with a recent study by Piers et al. [28]. Piers et al. identified a “locked immunometabolic switch” in the same R47H homozygous iPSC line used in this manuscript, namely a defect in upregulating glycolytic energy production in response to sudden energy demands placed on the cell, for example proinflammatory stimulation [28]. Cytokine secretion, phagocytosis, and migration require the cells to alter their metabolism rapidly, increasing glycolysis to supply energy. Importantly, metabolic defects in R47H cells were uncovered using manipulations with LPS + IFNγ and glycolysis inhibitors, whereas the cells were similar to the isogenic control when unstimulated [28]. This suggests that pathogenic manifestation of the mutation requires cell stress or high levels of activation. In contrast to our findings, a study of human R47H TREM2 expressed in a mouse tauopathy revealed striking attenuation of microgliosis and reduced phagocytosis of synaptic elements by microglia [69]. The authors also measured significantly lower levels of phagocytosed synapse material in microglia of human AD brains with R47H/R62H TREM2 variant, versus case-matched AD brains with TREM2 common variant. The reduction in synapse phagocytosis mirrors the effect of TREM2 KO in our data and in the literature. This provides further support to our hypothesis that in a “disease” setting, the R47H TREM2 mutation causes measurable phenotypic defects that reflect a reduction of TREM2 function. Our in vitro model recreates healthy isolated microglia, and under these conditions, the R47H TREM2 cells did not exhibit significant defects of synaptosome phagocytosis.

### Basis of divergent cell phenotypes of R47H TREM2 and TREM2 KO

In the absence of overt phenotypic defects in the R47H TREM2 pMac, we turned to transcriptomics to uncover any “hidden” dysfunction. Contrasting with the divergent physical phenotypes, there was high overlap between DEGs identified in R47H TREM2 pMac and TREM2 KO. With very few dysregulated genes that are unique to the R47H TREM2 pMac, it is unlikely that these can explain the major differences in phenotype between the R47H and KO lines. The largest discrepancy is the gene *CHCHD2*, which is transcriptionally upregulated 21-fold in TREM2 KO and downregulated 153-fold by R47H TREM2 relative to the WT (Fig. 5a; Additional file 1: Fig. S8). Loss of function mutations of *CHCHD2* are linked to autosomal dominant Parkinson’s disease, and *CHCHD2* encodes a protein proposed to have multiple roles as a mitochondrial ROS scavenger, scaffold, inhibitor of apoptosis, and transcription factor, collectively promoting activity and stability of the mitochondrial respiratory chain in response to mitochondrial stressors [70–72]. The only gene known to be directly regulated by CHCHD2 is *COX4I2*, which was not differentially expressed in TREM2 KO or R47H, so it is not clear whether CHCHD2 dysregulation in R47H or KO has biological relevance in pMac. A more likely explanation for the absence of phenotypic defects in the R47H line is that less than a third of the genes affected in the KO were significantly dysregulated, with the degree of upregulation or downregulation frequently intermediate to the KO (Figs. 4c and 5a). Although transcriptional dysregulation is evident in unstimulated R47H pMac, there may be sufficient tonic TREM2-DAP12 activity under standard cell culture conditions to avoid overt phenotypic defects. Furthermore, there is a degree of redundancy in the regulation of important microglia functions. For example, we demonstrated that TREM2 KO only has a detrimental effect on long-term survival when M-CSF/CSF1 is removed from the media (Fig. 3e–f). Standard cell culture conditions include excess M-CSF/CSF1, and CSF1 receptor signalling converges on similar downstream mediators to TREM2, potentially mitigating the impact of TREM2 loss of function on multiple phenotypes.

### Previous transcriptomic studies of TREM2 KO and R47H TREM2

The transcriptional effects of *TREM2* knockout have been previously studied in mouse models [73–77] and human stem cell-derived microglia-like cells [11, 27]. Our study is consistent with two mouse studies that demonstrated enrichment of pathways relating to inflammatory and cytokine responses, and chemotactic motility [45, 73]. On the other hand, genes identified in mice to be “homeostatic” are highly upregulated in Trem2 KO mouse microglia relative to WT, such as *P2ry12*, *Mertk*, and *Tmem119*, which are not significantly altered in our human TREM2 KO pMac. A third Trem2 KO mouse study primarily found enrichment for unfolded protein response and protein ubiquitination [74]. Unfolded protein response was not significantly enriched in our TREM2 KO transcriptome, but the protein ubiquitination was enriched in PPI “group 3” for TREM2 KO. Species-specific differences may account for some discrepancy of our transcriptome with the mouse studies. Zhou et al. (2020) observed that a microglial “disease-associated” transcriptional signature in human AD brain was distinct from that identified in mouse AD models previously, with very few genes in common [78]. In TREM2 KO human stem-cell-derived microglia-like cells, two independent transcriptomic analyses have been performed previously in the literature [11, 27]. Comparing significant DEGs identified by Claes et al. [27] with our own, there is significant correlation (Additional file 1: Fig. S11). Considering the distinct differentiation protocol and cell line used in Claes et al. [27], this finding is encouraging. The top GO pathways identified in the Claes et al. study are consistent with our study, with upregulation of cytokine/chemokine activity and calcium ion binding, and downregulation of extracellular matrix structure and integrins [27]. Andreone et al. [11] specifically examined dysregulated genes shared by both TREM2 KO and PLCG2 KO iPSC-microglia-like cells, therefore a subset of TREM2 KO DEGs, and found significant enrichment for DAP12-mediated signalling, phagocytosis of pathogens, and complement system in TREM2 KO. We did not find that these were highly enriched GO pathways in our whole TREM2 KO transcriptome, but noted significant upregulation of some key genes associated with these processes, including *SYK*, *PLCG2*, *FCGR1B/C*, *CR1*, *C3AR1*, and *C5AR1/2*.

### TGFβ1 in the context of microglia function and AD

We identified potential chemical and biologic agents that could reverse transcriptional dysregulation of the TREM2 KO (and by implication R47H TREM2). Interestingly, the immunosuppressive steroid drug, dexamethasone, was the strongest predicted upstream regulator opposing TREM2 KO (Table 2). The effects of dexamethasone on TREM2 signalling pathways will be followed up in subsequent work. In our current study, we investigated TGFβ1, the second highest hit in Table 2. TGFβ1 is a growth factor crucial for maintaining microglial “identity” of murine primary microglia after removal from the brain [79] and is used in some differentiation protocols for iPSC-microglia-like cells [42]. However, it is not supplemented in our differentiation protocol. Our iPSC-macrophages secrete TGFβ1 in culture, at similar levels to previously published rat microglia [80]. Secretion is SYK-dependent, and TREM2 KO pMac express reduced levels of TGFβ1. The assay that we used for TGFβ1 does not discriminate between inactive and active TGFβ1; therefore, further work would be needed to confirm that these differences translate to a change in TGFβ receptor activation. Supplementation of cultures with TGFβ1 for 24 h resulted in increased expression of the vitronectin receptor (αVβ3 integrin) and appeared to promote αVβ3-dependent cell adhesion to vitronectin (Fig. 6e–f). Interestingly, TGFβ1 has long been linked to AD in the literature. There is evidence of increased TGFβ1 in cerebrospinal fluid of AD patients; however, downstream SMAD-dependent signalling of TGFβ receptors is hypothesised to be dysfunctional in AD [81]. In mouse AD models, stimulating TGFβ1 levels by various pharmacological means is neuroprotective and may be partly mediated by a reduction in microglial inflammation and increased degradation of Aβ by microglia [81]. We aimed to validate a bioinformatic prediction, rather than demonstrate the therapeutic usefulness of TGFβ1, which has been done by others (e.g., references [82, 83]). TGFβ1 promoted vitronectin receptor expression as predicted; however, this did not fully rescue the defective vitronectin adhesion of R47H TREM2 and TREM2 KO pMac. We speculate that vitronectin adhesion requires additional surface molecules such as uPAR that may be downregulated in R47H and TREM2 KO pMac.

## Conclusions

In a model of human microglia, we show that the R47H TREM2 risk variant for AD has similar transcriptional dysregulation to TREM2 KO, but does not share deficits of phagocytosis, chemotaxis, and survival that manifest in the TREM2 KO cells. Transcriptomics shone a light on specific defects in adhesion that are shared by R47H TREM2 and TREM2 KO cells. We additionally predicted that TGFβ1 may alleviate some deficits caused by the TREM2 KO; however, the effect of TGFβ1 on cell adhesion was limited. Given that the R47H mutation leads to a 2- to 4.5-fold increased risk of AD [18, 20], it is worth using the transcriptomic information to continue to identify microglial phenotypes affected by the risk allele, and potential therapeutic targets.

## Supplementary information

**Additional file 1 :** Figure S1. Validation of R47H genotype. (A) CRISPR single guide RNA used for insertion of R47H mutation by Bioneer. (B) Chromatograms from sequencing of WT line BIONi010-C and R47H TREM2 line BIONi010-C-7. Red asterisk indicates the R47H mutation, black asterisks are silent mutations added by Bioneer to prevent re-cutting. Figure S2. SNP microarray of iPSCs. Chromosome karyograms from Illumina microarray SNP analysis, showing (A) BIONi010-C line, (B) BIONi010-C-7 R47H TREM2 line, (C) BIONi010-C-17 TREM2 KO line. Figure S3. Validation of R47H TREM2 and TREM2 KO pMac. (A) Macrophage surface markers CD11b, CD14, and CD45 measured by flow cytometry. Median fluorescence intensity (MFI) for each sample was normalized to the relevant isotype IgG, and then to the average for the three genotypes. Histogram shows means ± SEM, for *n*=3-4 harvests. 1-way ANOVA with Dunnett’s post-hoc test, comparisons to WT line. ** *p* < 0.01, *** *p* < 0.001, **** *p* < 0.0001, all unannotated comparisons are not significant. (B) Total levels of TREM2 protein shown in a representative western blot (WB). (C-D) Surface TREM2 measured by immunofluorescence staining (IF): live pMac were stained with TREM2 antibody, followed by fluorescent secondary antibody, and subsequently fixed. Images are maximum projections from a z-stack of 5 slices, 1-5 μm, taken on an Opera Phenix microscope (Perkin Elmer). Quantified mean fluorescence (per μm^2^), for triplicate wells, was normalised to the average for the three genotypes, and then expressed as a ratio of whole-cell TREM2 staining from separate permeabilised wells on the same plate (D). Means ± SEM, for *N*=3 harvests, *p* = 0.047 in one-tailed paired t-test. (E-F) Kinetics of pMac calcium responses to 0.5 mM ATP (E), and 10 μg/mL TREM2 antibody (F). Means ± SEM, for N=3-5 harvests. Figure S4. Validation of antibodies for TREM2 immunocytochemistry. Fixed and permeabilized WT, R47H, and TREM2 KO pMac were stained for 1 hour at RT with three different TREM2 antibodies at the concentrations indicated, followed by staining with Alexa Fluor 488-conjugated secondary antibody (1:1000, Invitrogen). Cells were counterstained with DAPI nuclear dye and imaged on an EVOS FL Auto automated microscope (Thermo Fisher). Ab209814 showed cytoplasmic staining in all three genotypes, 13,483–1-AP showed nuclear staining in all three genotypes, whereas AF1828 stained cytoplasm and plasma membrane in WT and R47H TREM2 pMac but not TREM2 KO pMac. Scale bar is 100 μm. Figure S5. Validation of dead SH-SY5Y phagocytosis assay. (A) Freshly-fixed SH-SY5Ys stain uniformly for phosphatidylserine exposure (annexin V-FITC), but have limited cell permeability (propidium iodide). Live SH-SY5Ys do not stain for annexin V-FITC or propidium iodide, except for focal staining present on the few dead cells in culture. (B) No TREM2 expression in an SH-SY5Y not undergoing phagocytosis, marked with a white arrow. (C) No RAB9 expression in non-engulfed SH-SY5Ys, marked with a white arrow. (D) Dose-dependent uptake of dead SH-SY5Ys after 5 hours of phagocytosis with WT line BIONi010-C, means quantified from three independent experiments for % of spot positive (phagocytic) cells per well. Means ± SEM, for N=3 harvests. (E) Phagocytosis of 3 hours is inhibited with 10 μM cytochalasin D, 1 μM bafilomycin A1, 1 μM jasplakinolide, all with 1 hour pre-treatment, and 13 μg/mL recombinant annexin V added simultaneously to the dead SH-SY5Ys. Data was normalized to mean for each genotype per experiment. Means ± SEM, for N=3-6 harvests and with two WT cell lines (SFC840-03-03, the characterisation of this line is described in Fernandes et al [32], and BIONi010-C). 1-way ANOVA with Dunnett’s post-hoc test, comparisons to untreated cells. * *p* < 0.05, *** *p* < 0.001. Figure S6. Validation of synaptosome phagocytosis assay. (A) Two whole synaptosomes surrounded by cell debris in the cryopreserved prep, visualised by negative staining electron microscopy. White asterisks label the pre-synaptic termini, with many pre-synaptic vesicles, whereas purple asterisks label the post-synaptic termini. A dark post-synaptic density can be seen between connected pre- and post-synaptic termini. (B) Synaptosomes stain uniformly for phosphatidylserine exposure (annexin V-FITC), comparison is with unstained synaptosomes. An area magnified by 5X is shown inset. (C) Dose-dependent uptake of dead SH-SY5Ys after 3 hours of phagocytosis with WT line BIONi010-C, reaching saturation above 30 μg. (D) Phagocytosis in BIONi010-C pMac is inhibited by 10 μM cytochalasin D and 1 μM bafilomycin A1, and increased by prior opsonisation of synaptosomes for 30 minutes with 20% human serum. Data was normalized to mean for each genotype per experiment, and is represented as sum of spot areas (μm^2^) per cell. Means ± SEM, for N=3-4 harvests. 1-way ANOVA with Dunnett’s post-hoc test, comparisons to untreated cells. * p < 0.05, ** *p* < 0.01. Figure S7. Validation for cytokine ELISAs and transwell chemotaxis assay. Cytokine ELISAs: (A) Secretion of TNF in response to 4 hours of 0.1-1 μg/mL LPS. (B) Secretion of IL-6 in the same supernatants as (A). Means ± SEM, for N=3 harvests. 2-way ANOVA with Dunnett’s post-hoc test. Comparisons with the coloured annotations are stimulations versus untreated cells (None) for each genotype. Comparisons with the black annotations are R47H or KO versus the WT line for each stimulation, all unannotated comparisons are not significant. Transwell chemotaxis assay: (C) Migration of WT pMac in transwell chemotaxis assay in the presence of four concentrations of ADP or C5a, for 6 hours. (D) Migration of WT pMac for 6 hours in the presence of 30 μM ADP is attenuated by 30 minutes pre-treatment with a P2RY12-selective inhibitor (PSB0739), but not a P2RY1 (MRS2179) or P2RY13 (MRS2211) inhibitor. (E) Migration of WT pMac for 6 hours in the presence of 3 nM C5a is attenuated by 30 minutes pre-treatment with a C5aR inhibitor (PMX-53), or a Syk inhibitor (OXSI-2, 3 μM). Means ± SEM, for N=3-4 harvests. 1-way ANOVA with Dunnett’s post-hoc test, pairwise comparisons to control. * p < 0.05, ** p < 0.01, *** p < 0.001, **** *p* < 0.0001. Figure S8. RNA-seq differentially-expressed genes (DEGs). Volcano plots shown for DEGs relative to WT: (A) TREM2 KO and (B) R47H TREM2 pMac. Dashed lines show cut-offs at log2-fold-change=2 and *p*=0.001. Enrichment of Gene Ontology (GO) terms in significant (adjusted *p* value <0.05) DEGs relative to WT: (C) TREM2 KO, and (D) R47H TREM2 pMac. Top 30 terms shown in order of the adjusted p value, the relative R–score represents the average direction of change. Circle size corresponds with number of DEGs. Figure S9. Flow cytometry of integrins. Representative flow cytometry histograms for Figure 6e. Figure S10. Proportions of TREM2 transcript splice variants. TREM2 differential transcript usage analysis was performed on the RNA-seq data with the DRIMSeq bioconductor package, using Salmon-quantified transcript counts. DRIMSeq assumes an Dirichlet Multinomial model (DM) for each gene, where the total count for the gene is considered fixed, and the quantity of interest is the proportion for the transcript within a gene for each sample. A likelihood ratio test was used to test for gene and transcript level DTU between R47H vs WT, and no significant difference in transcript usage was found. Transcripts are described in Del-Aguila et al [62]: ENST00000373113 is the canonical TREM2 transcript and the longest, with five exons; ENST00000373122 is the second longest and lacks the 5’ exon but includes the transmembrane domain; ENST00000338469 is the shortest and excludes the transmembrane domain. The bars represent three sequential ages of iPSC-macrophages. Figure S11. Correlation of TREM2 KO DEGs with Claes et al (2019). Log-2-fold change values for the significant DEGs (adjusted p value <0.05) of the current study (purple) plotted against significant DEGs from Claes et al (orange). Overlapping significant DEGs shown in green.

**Additional file 2.** : Video 1. Time-lapse video of dead SH-SY5Y phagocytosis assay. WT pMac phagocytosing pHrodo-labelled fixed SH-SY5Ys, displaying an increase in red fluorescence of the SH-SY5Ys after engulfment. In 96wp, 2 x 10^4^ pMac were given 2 x 10^4^ SH-SY5Ys, and images taken every 10 minutes at 20x for 3 hours, using an EVOS FL Auto automated microscope. Video is 2 frames per second.

**Additional file 3.** : Video 2. Time-lapse video of synaptosome phagocytosis assay. WT pMac phagocytosing pHrodo-labelled synaptosomes, displaying an increase in red fluorescence of the synaptosomes after engulfment. In 96wp, 2 x 10^4^ pMac were given 1 μg of synaptosomes, and images taken every 10 minutes at 20x for 3 hours, using an EVOS FL Auto automated microscope. Video is 2 frames per second.

**Additional file 4 **Results of RNA-seq differential gene expression analysis with DESeq2. DESeq2 output for R47H TREM2 vs WT and TREM2 KO vs WT, with gene name, relative expression (“Base Mean”), log-2-fold-change from the WT, and Benjamini-Hochberg-adjusted *p* values indicated.

**Additional file 5.** Results of RNA-seq Ingenuity Pathways Analysis on TREM2 KO PPI groups. For each PPI group separate files give information about the genes assigned to that group (suffix “_Genes”), the predicted upstream regulators with Activation Z-score indicating whether the change mimics (+) or opposes (−) TREM2 KO transcriptional effects (suffix “_UpstreamAnalysis”), and the relationship of predicted upstream regulators to disease/functions (suffix “_RegulatorEffects”).

## Data Availability

Data generated or analysed during this study are included in this published article and its supplementary information files, apart from the RNA-seq dataset, which is deposited in NCBI’s Gene Expression Omnibus (Edgar et al., 2002) and is accessible through GEO Series accession number GSE157635 (https://www.ncbi.nlm.nih.gov/geo/query/acc.cgi?acc=GSE157635)..

## Abbreviations

KO: Knockout
iPSC: Induced pluripotent stem cell
pMac: iPSC-macrophage
LPS: Lipopolysaccharide
PtdSer: Phosphatidylserine
RT: Room temperature
