# Supplementary material for: TREM2 Alzheimer’s variant R47H causes similar transcriptional dysregulation to knockout, yet only subtle functional phenotypes in human iPSC-derived macrophages
Source: Alzheimers Res Ther. 2020 Nov 16;12:151. doi: 10.1186/s13195-020-00709-z (PMC7667762; doi:10.1186/s13195-020-00709-z)
Supplement: Supplementary file 1 — Additional file 1 : Figure S1. Validation of R47H genotype. (A) CRISPR single guide RNA used for insertion of R47H mutation by Bioneer. (B) Chromatograms from sequencing of WT line BIONi010-C and R47H TREM2 line BIONi010-C-7. Red asterisk indicates the R47H mutation, black asterisks are silent mutations added by Bioneer to prevent re-cutting. Figure S2. SNP microarray of iPSCs. Chromosome karyograms from Illumina microarray SNP analysis, showing (A) BIONi010-C line, (B) BIONi010-C-7 R47H TREM2 line, (C) BIONi010-C-17 TREM2 KO line. Figure S3. Validation of R47H TREM2 and TREM2 KO pMac. (A) Macrophage surface markers CD11b, CD14, and CD45 measured by flow cytometry. Median fluorescence intensity (MFI) for each sample was normalized to the relevant isotype IgG, and then to the average for the three genotypes. Histogram shows means ± SEM, for n=3-4 harvests. 1-way ANOVA with Dunnett’s post-hoc test, comparisons to WT line. ** p < 0.01, *** p < 0.001, **** p < 0.0001, all unannotated comparisons are not significant. (B) Total levels of TREM2 protein shown in a representative western blot (WB). (C-D) Surface TREM2 measured by immunofluorescence staining (IF): live pMac were stained with TREM2 antibody, followed by fluorescent secondary antibody, and subsequently fixed. Images are maximum projections from a z-stack of 5 slices, 1-5 μm, taken on an Opera Phenix microscope (Perkin Elmer). Quantified mean fluorescence (per μm2), for triplicate wells, was normalised to the average for the three genotypes, and then expressed as a ratio of whole-cell TREM2 staining from separate permeabilised wells on the same plate (D). Means ± SEM, for N=3 harvests, p = 0.047 in one-tailed paired t-test. (E-F) Kinetics of pMac calcium responses to 0.5 mM ATP (E), and 10 μg/mL TREM2 antibody (F). Means ± SEM, for N=3-5 harvests. Figure S4. Validation of antibodies for TREM2 immunocytochemistry. Fixed and permeabilized WT, R47H, and TREM2 KO pMac were stained for 1 hour at RT with three differe [file 13195_2020_709_MOESM1_ESM.zip › Figure S6.pdf]

Additional file 1

Supplemental Data Fig S6. [Related to Fig.2]

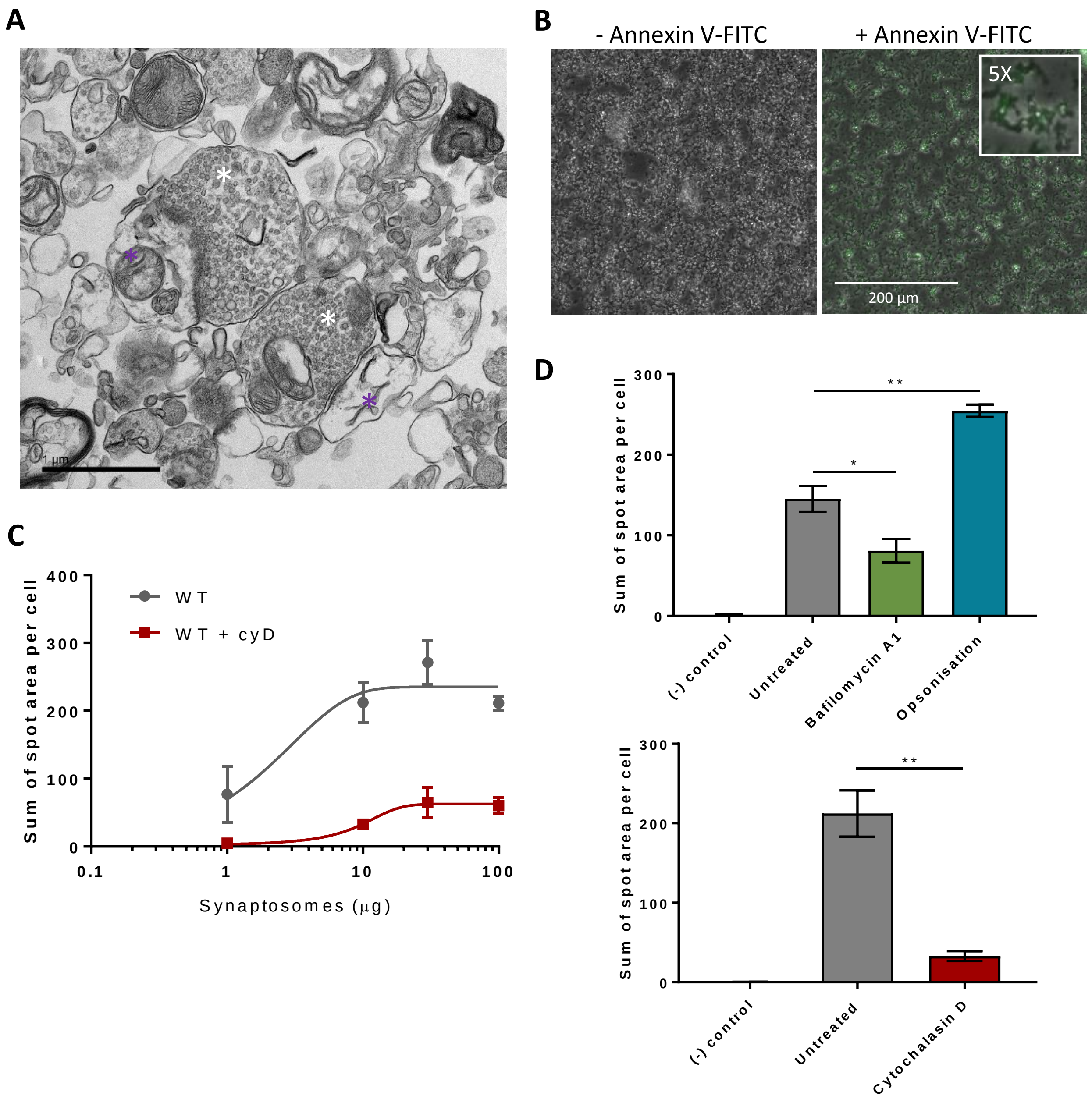

Figure S6. **Validation of synaptosome phagocytosis assay.**

(A) Two whole synaptosomes surrounded by cell debris in the cryopreserved prep, visualised by negative staining electron microscopy. White asterisks label the pre-synaptic termini, with many pre-synaptic vesicles, whereas purple asterisks label the post-synaptic termini. A dark post-synaptic density can be seen between connected pre- and post-synaptic termini. (B) Synaptosomes stain uniformly for phosphatidylserine exposure (annexin V-FITC), comparison is with unstained synaptosomes. An area magnified by 5X is shown inset. (C) Dose-dependent uptake of dead SH-SY5Ys after 3 hours of phagocytosis with WT line BIONi010-C, reaching saturation above 30 µg. (D) Phagocytosis in BIONi010-C pMac is inhibited by 10 µM cytochalasin D and 1 µM bafilomycin A1, and increased by prior opsonisation of synaptosomes for 30 minutes with 20% human serum. Data was normalised to mean for each genotype per experiment, and is represented as sum of spot areas (µm^2) per cell. Means ± SEM, for N=3-4 harvests. 1-way ANOVA with Dunnett's post-hoc test, comparisons to untreated cells. * $p < 0.05$, ** $p < 0.01$.
